# The burden and trend prediction of atrial fibrillation and flutter associated with lead exposure: insights from the global burden of disease study 2021

**DOI:** 10.3389/fcvm.2025.1638747

**Published:** 2025-08-12

**Authors:** Lili Deng, Qinhong Li, Hongyan Li, Bin Li, Zugen Cheng, Ying Xiao

**Affiliations:** ^1^ Department of Cardiology, Kunming Children’s Hospital, Kunming, China; ^2^ Department of Cardiology, The First Affiliated Hospital of Kunming Medical University, Kunming, China; ^3^ Department of Cardiology, Children’s Hospital Affiliated to Kunming Medical University, Kunming, China

**Keywords:** atrial fibrillation and flutter, lead exposure, global burden of disease study (2021), mortality, DALYs—disability-adjusted life years

## Abstract

**Background:**

Atrial fibrillation and flutter (AF/AFL) are increasingly recognized as major contributors to global cardiovascular morbidity and mortality. Emerging evidence implicates environmental lead exposure as a modifiable risk factor for AF/AFL, yet the global burden and trends of AF/AFL attributable to lead exposure remain poorly characterized.

**Methods:**

We used data from the Global Burden of Disease Study 2021 to estimate mortality, disability-adjusted life years (DALYs), and temporal trends in AF/AFL attributable to lead exposure from 1990 to 2021, with projections to 2030. Analyses were stratified by age, sex, and Socio-demographic Index (SDI) quintiles. Population-attributable fractions were calculated using comparative risk assessment methodology. Trend analyses utilized Joinpoint regression, and projections applied BAPC models.

**Findings:**

Between 1990 and 2021, the global burden of AF/AFL attributable to lead exposure increased substantially. The number of lead-attributable AF/AFL deaths rose by 264.9%, and DALYs increased by 179.3%. Age-standardized rates for mortality and DALYs rose by 30.7% and 16.3%, respectively. The highest attributable burden occurred in older adults (≥60 years) and low-SDI regions, where lead exposure remains pervasive. Males consistently exhibited higher AF/AFL mortality and DALY rates than females, although the sex gap is narrowing. A significant negative correlation was observed between SDI and both mortality (*r* = –0.53, *p* < 0.001) and DALY rates (*r* = –0.53, *p* < 0.001) for lead-attributable AF/AFL. Projections indicate a continued rise in global AF/AFL burden linked to lead exposure in the absence of further mitigation efforts.

**Interpretation:**

Lead exposure is an important, preventable contributor to the rising global burden of AF/AFL, particularly among older adults and populations in low-SDI regions. Mechanistically, lead may increase AF/AFL risk through both direct myocardial effects and the amplification of established cardiovascular risk factors, notably hypertension. Our findings support urgent global policy action to reduce environmental lead exposure as an integral strategy for cardiovascular and arrhythmia prevention.

## Introduction

Atrial fibrillation and flutter (AF/AFL) are the most common sustained cardiac arrhythmias globally and pose a substantial public health challenge (1). These arrhythmias markedly impair quality of life and elevate the risk of serious complications, including ischemic stroke (2), heart failure (3), and all-cause mortality (4). The global burden of AF/AFL has risen dramatically in recent decades. By 2019, an estimated 59.7 million people were living with AF/AFL worldwide—nearly double the number in 1990 (5). In the same year, AF/AFL was responsible for roughly 8.4 million disability-adjusted life-years (DALYs) lost and about 320,000 deaths globally (6), underscoring its significant contribution to cardiovascular morbidity and mortality. In short, AF/AFL remains a common and consequential cardiovascular disorder with an expanding global footprint that is projected to grow further as populations age and risk factor profiles worsen.

Lead exposure, meanwhile, is a pervasive environmental health threat with well-documented multisystem effects. Although early recognition of lead's hazards led to phasedown of leaded gasoline and other uses (culminating in a global ban on leaded petrol by 2021 (7), human exposure to lead remains widespread via other sources—including legacy lead-based paints, contaminated soil and dust, unsafe recycling of lead-acid batteries, and ingestion of lead-laced food or water (8). As a result, lead continues to exact a large toll on health worldwide. In 2019, an estimated 0.9–1.0 million deaths were attributable to lead exposure globally (7), accounting for roughly half of all deaths from known chemical exposures that year (9). Even at blood lead concentrations previously considered “low,” epidemiological studies have found associations with hypertension, coronary artery disease, and heart failure (10–12). Thus, despite decades of progress in reducing overt lead poisoning, chronic low-level lead exposure remains a significant global health burden—and importantly, one that is largely preventable.

Against this backdrop, there is growing interest in the potential linkage between lead exposure and AF/AFL—two public health challenges that until recently have been studied mostly in isolation. However, major knowledge gaps persist at the intersection of AF/AFL and lead exposure. To our knowledge, no prior study has comprehensively examined the global burden of AF/AFL attributable to lead exposure, nor projected how this burden might evolve over time. While global analyses have characterized AF/AFL trends and risk factors in general, the specific contribution of an environmental toxicant like lead has not been a focal point. This study aims to provide the first comprehensive global assessment of AF/AFL burden attributable to lead exposure, using Global Burden of Disease (GBD) 2021 data, and to project future trends by age, sex, and sociodemographic development.

## Methods

### Overview and methodological details

The GBD data source is recognized as one of the most comprehensive and systematic global epidemiological initiatives. Led by the Institute for Health Metrics and Evaluation (IHME) at the University of Washington, its objective is to quantify health losses attributable to various diseases, injuries, and risk factors (13). The GBD framework enables comparative assessments of morbidity and mortality across countries, regions, and globally.

Two key metrics were employed in GBD analyses to quantify disease burden: mortality and DALYs. DALYs represent the sum of years of life lost (YLL) due to premature mortality and years lived with disability (YLD) (14). The YLL component is calculated using Equation (1):

(1) $$\begin{array}{rl} & Y\mathrm{LL}\;=\\ & \;\mathrm{Number}\;\mathrm{of}\;\mathrm{deaths}\;\;\times \;\mathrm{Standard}\;\mathrm{life}\;\mathrm{expectancy}\;\mathrm{at}\;\mathrm{the}\;\mathrm{age}\;\mathrm{of}\;\mathrm{death} \end{array}$$

while YLD is derived from Equation (2):

(2) YLD=Diseaseprevalence×Disabilityweight

This study retrieved and analyzed data on AF/AFL-related mortality and DALYs attributable to lead exposure from 1990 to 2021. Data were sourced from the GBD database (https://vizhub.healthdata.org/gbd-results/). The GBD data used in our analysis were downloaded on April 3, 2024. The analysis incorporated multiple dimensions, including sex, age, and geographic location. However, no race- or ethnicity-related analyses were performed due to the unavailability of such parameters in the GBD database.

This cross-sectional study involved the analysis and description of disease data across different time periods and regions without including identifiable personal information. Consequently, the Ethics Committee of Kunming Children's Hospital granted an exemption from informed consent procedures. The study strictly adhered to the Strengthening the Reporting of Observational Studies in Epidemiology (STROBE) guidelines (15).

### Definition and diagnosis of AF/AFL

AF and AFL cases in the GBD study are identified based on standardized case definitions. Diagnosis is established through documented medical records, including either surface 12-lead electrocardiogram (ECG) or ambulatory Holter ECG findings consistent with AF or typical/atypical AFL, in accordance with international guidelines (e.g., ICD-10: I48). The GBD study harmonizes diagnostic criteria across data sources, but relies on the best available data in each country; thus, the mode of diagnosis may vary between countries and regions depending on local clinical practice and surveillance systems. Further details on AF/AFL diagnostic ascertainment are described in the GBD 2021 study protocol (13).

### Definitions of lead exposure

In the GBD framework, lead exposure is quantified primarily through population-level blood lead concentration data from published surveys, national health databases, and environmental models. The standard GBD exposure metric represents the population-weighted mean blood lead concentration (μg/dl) for each country-year-age-sex stratum, with 0 μg/dl designated as the theoretical minimum risk exposure level (TMREL). Relative risk estimates follow a continuous exposure–response relationship, derived from meta-analyses of epidemiological studies. Since blood lead measurements were unavailable for all individuals, GBD integrates available national and regional survey data with predictive modeling to address gaps. Measurement methodologies and analytical techniques, including atomic absorption spectrometry and inductively coupled plasma mass spectrometry, are detailed in the GBD 2021 risk factors protocol (16).

In our study, lead exposure was defined as the extent to which individuals were exposed to lead in daily life, originating from contaminated water, airborne lead particles, and soil lead levels. Lead exposure can be classified into two types: acute and chronic. Acute lead exposure, measured in micrograms per deciliter of blood (μg/dl), was associated with impaired intellectual development in children. Conversely, chronic lead exposure, quantified in micrograms of lead per gram of bone (μg/g), was directly correlated with increased systolic blood pressure and elevated risk of cardiovascular diseases. The U.S. Centers for Disease Control and Prevention (CDC) has established blood lead reference values of 3.5 μg/dl for children and 5 μg/dl for adults, with the 2021 update revising the reference value from 5 μg/dl to 3.5 μg/dl (17, 18).

### SDI

The SDI is a comprehensive metric for evaluating the socioeconomic development of a country or region (19). This index encompasses multiple dimensions, including but not limited to economic structure and scale, educational attainment, living standards, as well as social welfare and security. The index value ranges from 0 to 1, with higher values indicating superior socioeconomic development. According to the GBD database, countries and regions are classified into five categories based on their SDI: low, low-middle, middle, high-middle, and high. This classification facilitates the investigation of the impact of socioeconomic indices and geographical disparities on the burden of AF/AFL attributable to lead exposure.

### Bayesian age-period-cohort (BAPC) analysis

The BAPC model is a methodological approach employed in epidemiology and biostatistics to analyze the temporal trends of mortality and DALYs associated with AF/AFL due to lead exposure. By integrating sample information and prior knowledge, this model yields unique parameter estimates, ensuring robust and reliable results (20–22).

### Statistical analysis

According to the GBD database, mortality and disability rates per 100,000 population were calculated along with their 95% uncertainty intervals (UIs). The Joinpoint regression model was employed to estimate the annual percentage change (APC) and its 95% confidence interval (CI) to assess temporal trends within each independent period (23). This method provides a detailed understanding of annual rate fluctuations, offering a granular perspective on year-to-year variations.

A log-transformed linear regression model was used to compute the estimated average annual percentage change (EAPC) and its CI for analyzing temporal trends in mortality and DALYs attributable to lead exposure-induced AF/AFL from 1990 to 2021 (24). The EAPC is particularly valuable for examining long-term trends, as it untangles whether rates generally increase or decrease over time, irrespective of short-term fluctuations. An EAPC value and the lower bound of its 95% CI greater than 0 indicate an upward trend in the corresponding metric. Conversely, an EAPC value and the upper bound of its 95% CI less than 0 suggest a downward trend.

The relationship between disease burden indicators and the SDI was analyzed using fitted curves. All statistical analyses in this study were performed using R software (version 4.4.2), with a significance threshold of *p* < 0.05.

## Result

### Global burden of AF/AFL associated with lead exposure

#### Mortality

Globally, mortality rates from AF/AFL attributable to lead exposure demonstrated an initial increase followed by a subsequent decline over the past three decades. The highest APC was observed between 1990 and 1995, reaching 2.10% (95% CI, 1.89–2.32). A peak in mortality was recorded in 2017, at 0.12 deaths per 100,000 population (95% UI, −0.02 to 0.30). The number of global deaths due to lead exposure-related AF/AFL increased from 2,480.89 (95% UI, −357.17 to 6,298.29) in 1990 to 9,053.18 (95% UI, −1,392.65 to 22,236.49) in 2021, representing an overall increase of 264.92% (95% UI, 217.93–326.84). Correspondingly, the mortality rate rose from 0.09 (95% UI, −0.01 to 0.22) per 100,000 in 1990 to 0.12 (95% UI, −0.02 to 0.29) per 100,000 in 2021, representing a 30.73% (95% UI, 15.07–50.79) increase. The EAPC was 0.90 (95% CI, 0.77–1.02).

**Figure 1:**
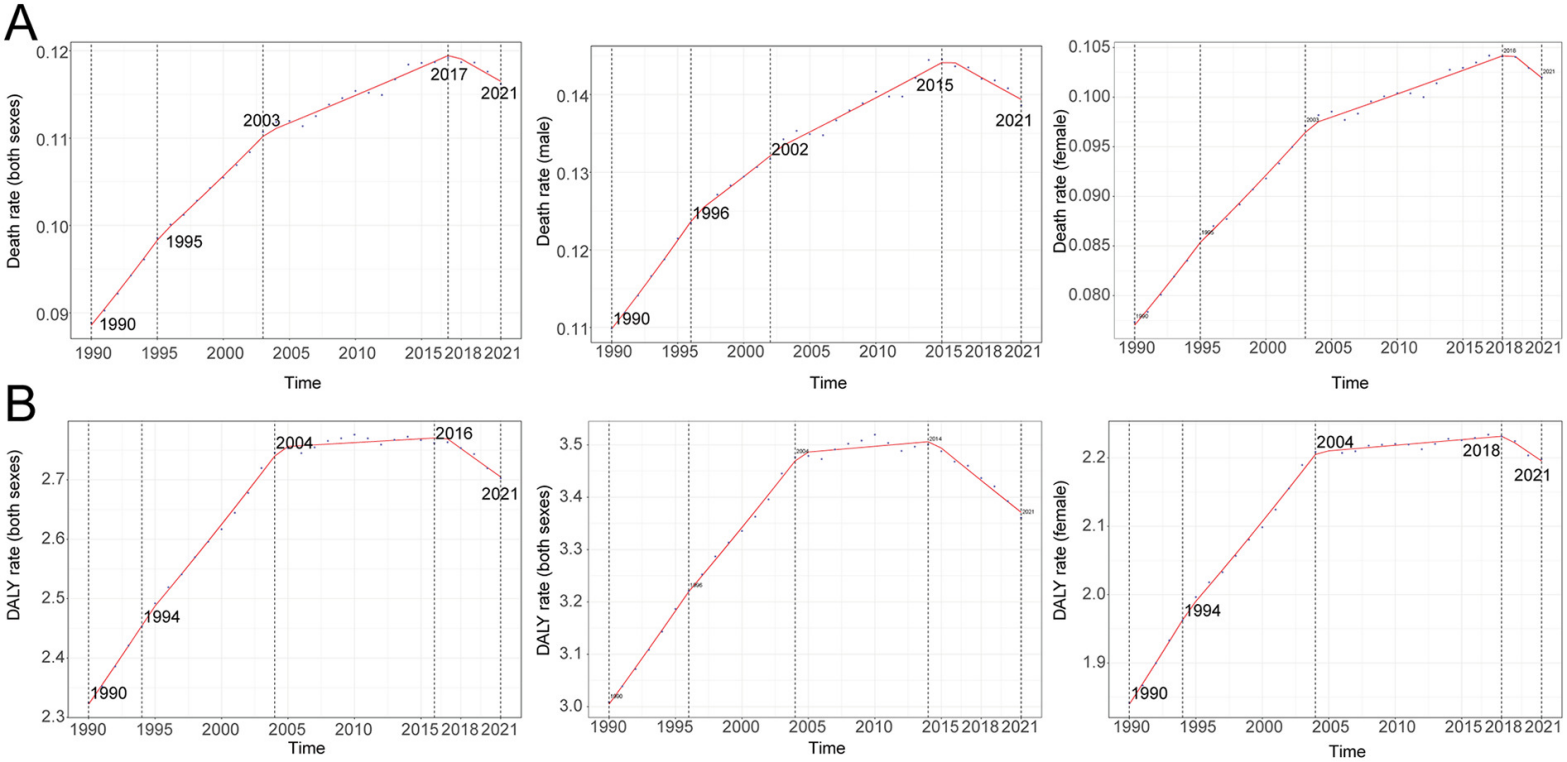
Annual percent change (APC) and trends in global atrial fibrillation and flutter mortality and disability-adjusted life years (DALYs) due to lead exposure from 1990 to 2021. **(A)** Mortality rate, **(B)** DALY rate.

**Figure 2:**
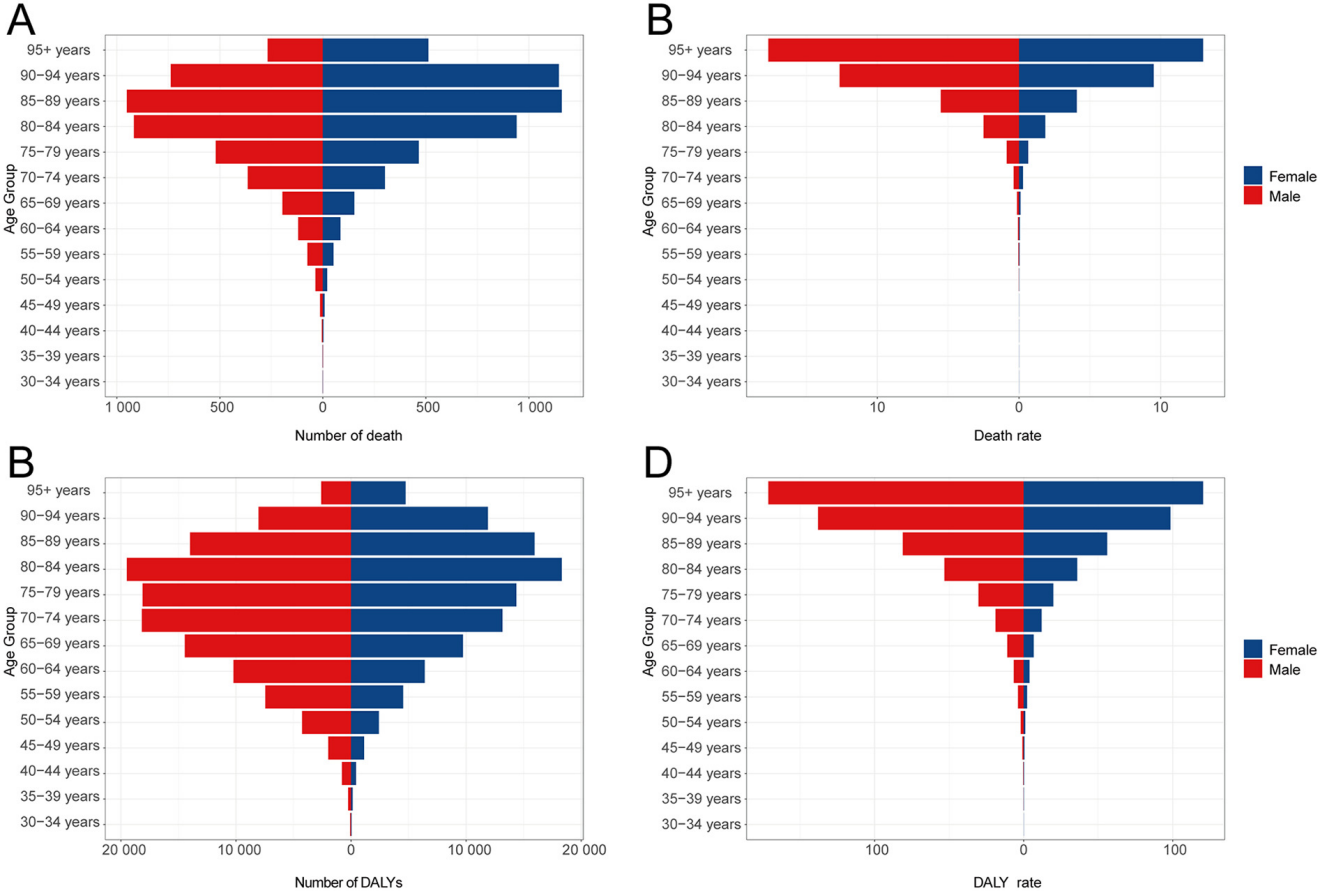
DALYs and deaths from atrial fibrillation and flutter due to lead exposure at different age groups in 2021. **(A)** Death cases, **(B)** Death rate, **(C)** DALY cases, **(D)** DALY rate.

Throughout the study period, mortality rates attributable to lead exposure were consistently higher among males compared to females (Table 1; Figure 1A). In 2021, age-specific analyses revealed that AF/AFL mortality related to lead exposure increased with advancing age, peaking in the 85–89 years group for both sexes (approximately 951 deaths among males and 1,159 among females). The highest age-specific mortality rates were observed in those aged 95 years and above—17.70 per 100,000 (95% UI, −2.79 to 44.40) for males and 12.99 per 100,000 (95% UI, −2.06 to 33.11) for females (Figures 2A,B).

**Table 1:** Mortality of lead exposure-related atrial fibrillation and flutter between 1990 and 2021 at the global and regional level.

Location	1990 (95% UI)		2021 (95% UI)		1990–2021		
	Death cases	Death rate	Death cases	Death rate	Cases change^b^	Rate change^b^	EAPC^a^
Global	2,480.89 (−357.17, 6,298.29)	0.09 (−0.01, 0.22)	9,053.18 (−1,392.65, 2,2236.49)	0.12 (−0.02, 0.29)	264.92 (217.93, 326.84)	30.73 (15.07, 50.79)	0.90 (0.77, 1.02)
High SDI	738.25 (−106.45, 1,834.60)	0.07 (−0.01, 0.17)	2,017.36 (−300.32, 5,033.64)	0.07 (−0.01, 0.18)	173.26 (148.00, 189.93)	6.26 (−1.15, 11.90)	0.28 (0.12, 0.44)
High-middle SDI	524.43 (−74.64, 1,323.41)	0.08 (−0.01, 0.19)	1,954.05 (−302.87, 4,956.26)	0.11 (−0.02, 0.27)	272.60 (213.77, 343.17)	36.77 (15.76, 62.01)	1.07 (0.86, 1.27)
Middle SDI	644.18 (−89.78,1,654.29)	0.12 (−0.02, 0.29)	2,751.05 (−434.48, 6,811.73)	0.14 (−0.02, 0.35)	327.06 (248.89, 428.67)	19.94 (−1.32, 46.82)	0.49 (0.34, 0.64)
Low-middle SDI	420.39 (−67.65, 1,141.14)	0.12 (−0.02, 0.32)	1,799.96 (−281.76, 4,662.29)	0.19 (−0.03, 0.49)	328.16 (247.22, 451.98)	57.06 (29.11, 99.88)	1.54 (1.43, 1.64)
Low SDI	150.26 (−23.48, 411.07)	0.13 (−0.02, 0.35)	521.94 (−82.57, 1,375.40)	0.19 (−0.03, 0.49)	247.35 (185.47, 357.45)	48.08 (22.14, 94.07)	1.57 (1.33, 1.82)
Regions							
Andean Latin America	17.98 (−2.64, 48.48)	0.11 (−0.02, 0.30)	60.72 (−8.49, 156.02)	0.11 (−0.02, 0.29)	237.71 (175.38, 328.06)	0.15 (−18.03, 27.01)	−0.15 (−0.32, 0.03)
Australasia	35.35 (−5.23, 88.23)	0.17 (−0.03, 0.43)	117.16 (−17.14, 296.31)	0.18 (−0.03, 0.45)	231.40 (194.50, 275.64)	2.47 (−8.59, 15.06)	0.30 (−0.01, 0.60)
Caribbean	37.82 (−5.43, 95.78)	0.19 (−0.03, 0.48)	106.80 (−16.15, 271.01)	0.19 (−0.03, 0.48)	182.38 (150.23, 223.59)	0.63 (−10.53, 14.86)	0.06 (−0.11, 0.22)
Central Asia	10.05 (−1.47, 26.92)	0.03 (−0.00, 0.07)	25.97 (−3.81, 63.88)	0.05 (−0.01, 0.11)	158.36 (113.67, 204.35)	64.48 (34.83, 95.73)	1.59 (1.37, 1.82)
Central Europe	85.72 (−12.08, 218.30)	0.07 (−0.01, 0.19)	191.51 (−27.70, 482.51)	0.08 (−0.01, 0.20)	123.41 (102.22, 147.76)	6.85 (−2.48, 18.61)	0.39 (0.09, 0.70)
Central Latin America	111.52 (−16.37, 276.52)	0.20 (−0.03, 0.49)	418.74 (−62.69, 1,052.01)	0.18 (−0.03, 0.46)	275.47 (239.52, 314.68)	−6.09 (−14.68, 3.62)	−0.29 (−0.41, −0.16)
Central Sub-Saharan Africa	11.12 (−1.63, 30.57)	0.10 (−0.01, 0.28)	39.53 (−6.17, 107.30)	0.14 (−0.02, 0.39)	255.47 (161.99, 363.86)	43.28 (7.00, 89.02)	1.18 (1.07, 1.30)
East Asia	523.68 (−73.03, 1,334.10)	0.14 (−0.02, 0.36)	2,356.55 (−382.39, 5,904.56)	0.15 (−0.02, 0.37)	350.00 (229.56, 513.64)	7.56 (−21.25, 46.52)	0.08 (−0.18, 0.33)
Eastern Europe	74.07 (−10.66, 194.33)	0.04 (−0.01, 0.09)	184.06 (−26.68, 454.19)	0.05 (−0.01, 0.12)	148.50 (114.74, 186.12)	44.73 (25.56, 66.25)	1.12 (0.92, 1.33)
Eastern Sub-Saharan Africa	50.65 (−6.96, 139.45)	0.13 (−0.02, 0.36)	119.03 (−17.52, 310.19)	0.13 (−0.02, 0.34)	135.03 (88.93, 202.22)	−0.01 (−18.23, 27.33)	−0.07 (−0.13, −0.00)
High-income Asia Pacific	70.51 (−9.87, 181.30)	0.04 (−0.01, 0.11)	271.49 (−40.77, 681.01)	0.04 (−0.01, 0.10)	285.06 (213.43, 334.72)	−10.09 (−26.65, −0.07)	−0.74 (−1.00, −0.48)
High-income North America	233.80 (−35.12, 573.91)	0.06 (−0.01, 0.16)	593.85 (−89.50, 1,516.97)	0.08 (−0.01, 0.20)	154.00 (129.10, 176.76)	21.94 (10.18,3 2.63)	0.71 (0.59, 0.82)
North Africa and Middle East	127.15 (−19.39, 323.17)	0.13 (−0.02, 0.33)	435.52 (−62.00, 1,085.80)	0.15 (−0.02, 0.39)	242.52 (164.56, 355.90)	19.29 (−7.00, 59.20)	0.71 (0.62, 0.81)
Oceania	0.81 (−0.12, 2.21)	0.06 (−0.01, 0.17)	2.51 (−0.39, 6.73)	0.06 (−0.01, 0.17)	208.72 (147.07, 276.51)	5.66 (−14.63, 27.25)	0.12 (0.06, 0.17)
South Asia	344.00 (−51.27, 941.02)	0.11 (−0.02, 0.29)	1,797.30 (−294.57, 4,572.32)	0.19 (−0.03, 0.48)	422.48 (293.59, 635.22)	74.48 (31.29, 145.99)	2.00 (1.80, 2.20)
Southeast Asia	118.68 (−18.04, 323.68)	0.08 (−0.01, 0.22)	482.89 (−71.88, 1,158.61)	0.11 (−0.02, 0.27)	306.88 (217.81, 416.71)	43.38 (12.14, 81.04)	1.06 (0.84, 1.28)
Southern Latin America	15.48 (−2.13, 39.23)	0.04 (−0.01, 0.11)	49.59 (−7.29, 124.46)	0.05 (−0.01, 0.13)	220.39 (189.23, 251.84)	27.54 (15.41, 39.84)	1.54 (1.14, 1.94)
Southern Sub-Saharan Africa	10.40 (−1.49, 27.08)	0.06 (−0.01, 0.15)	30.81 (−4.47, 76.09)	0.09 (−0.01, 0.21)	196.39 (132.40, 269.14)	54.24 (17.18, 96.75)	1.53 (1.09, 1.98)
Tropical Latin America	80.44 (−11.84, 197.22)	0.15 (−0.02, 0.36)	355.20 (−53.25, 890.40)	0.15 (−0.02, 0.38)	341.59 (305.51, 371.89)	2.71 (−4.07, 8.88)	0.07 (−0.09, 0.23)
Western Europe	466.32 (−67.48, 1,172.62)	0.08 (−0.01, 0.20)	1,271.53 (−189.67, 3,176.68)	0.10 (−0.01, 0.24)	172.67 (146.50, 190.77)	18.69 (9.32, 25.85)	0.79 (0.63, 0.96)
Western Sub-Saharan Africa	55.35 (−9.04, 150.11)	0.11 (−0.02, 0.31)	142.42 (−22.32, 362.12)	0.14 (−0.02, 0.35)	157.33 (104.09, 244.43)	21.24 (−3.01, 64.80)	0.42 (0.31, 0.52)

EAPC, estimated annual percentage change; SDI, sociodemographic index; UI, uncertainty interval.

^a^ EAPC is expressed as 95% confidence interval.

^b^ Change shows the percentage change.

#### DALYs

DALYs attributed to lead exposure mirrored those observed for mortality, with an overall pattern of initial increase followed by decline across the past three decades. The highest APC in DALY rates occurred from 1990 to 1994, at 1.39% (95% CI, 1.24–1.54). A peak in the DALY rate was observed in 2016 (2.76 per 100,000; 95% UI, −0.37 to 7.09). The global number of DALYs due to lead-related AF/AFL rose from 79,858.19 (95% UI, −10,305.86 to 205,458.67) in 1990 to 223,015.26 (95% UI, −29,502.69 to 563,551.70) in 2021—an increase of 179.26% (95% UI, 159.81–207.74). DALY rates increased from 2.32 (95% UI, −0.30 to 5.95) per 100,000 in 1990 to 2.70 (95% UI, −0.36 to 6.83) per 100,000 in 2021, an increase of 16.26% (95% UI, 8.04–27.07), with an EAPC of 0.50 (95% CI, 0.39–0.62).

DALY rates attributable to lead exposure remained consistently higher in males compared with females throughout the observation period (Table 2; Figure 1B). In 2021, DALY counts peaked in the 80–84 years group (19,481 for males and 18,306 for females), while the highest age-specific DALY rates were noted among those aged 95 years and above (171.35 per 100,000 [95% UI, −25.85 to 426.75 in males and 120.42 per 100,000 [95% UI, −18.43 to 303.45 in females) (Figures 2C,D).

**Table 2:** DALYs of lead exposure-related atrial fibrillation and flutter between 1990 and 2021 at the global and regional level.

Location	1990 (95% UI)		2021 (95% UI)		1990–2021		
	DALY cases	DALY rate	DALY cases	DALY rate	Cases change^b^	Rate change^b^	EAPC^a^
Global	79,858.19 (−10,305.86, 205,458.67)	2.32 (−0.30, 5.95)	223,015.26 (−29,502.69, 563,551.70)	2.70 (−0.36, 6.83)	179.26 (159.81, 207.74)	16.26 (8.04, 27.07)	0.50 (0.39, 0.62)
High SDI	20,311.70 (−2,620.37, 51,855.04)	1.84 (−0.24, 4.67)	43,024.71 (−5,724.91, 110,237.47)	1.79 (−0.24, 4.60)	111.82 (95.43, 128.74)	−2.29 (−8.38, 4.28)	−0.04 (−0.20, 0.11)
High-middle SDI	16,438.77 (−2,101.30, 42,274.86)	1.92 (−0.25, 4.87)	44,581.16 (−5,925.53, 112,242.19)	2.30 (−0.31, 5.80)	171.20 (146.59, 206.22)	20.09 (9.05, 33.41)	0.60 (0.42, 0.78)
Middle SDI	21,949.71 (−2,768.86, 55,497.47)	2.79 (−0.36, 7.10)	71,606.45 (−9,490.81, 180,990.07)	3.09 (−0.42, 7.77)	226.23 (194.67, 268.80)	10.83 (−1.09, 25.70)	0.29 (0.17, 0.42)
Low-middle SDI	15,658.73 (−2,136.54, 39,673.31)	3.33 (−0.46, 8.51)	48,642.02 (−6,668.18, 124,411.98)	4.23 (−0.59, 10.87)	210.64 (180.77, 253.14)	26.95 (14.28, 45.26)	0.81 (0.72, 0.89)
Low SDI	5,404.47 (−789.39, 14,115.63)	3.25 (−0.49, 8.58)	14,958.32 (−2,193.51, 37,534.19)	4.10 (−0.62, 10.39)	176.78 (148.58, 211.72)	26.35 (12.30, 43.32)	0.91 (0.80, 1.01)
Regions							
Andean Latin America	482.01 (−63.00, 1,262.53)	2.67 (−0.35, 6.99)	1,601.67 (−205.90, 4,102.58)	2.84 (−0.37, 7.29)	232.29 (193.24, 284.50)	6.58 (−6.19, 23.85)	0.16 (0.06, 0.25)
Australasia	886.76 (−119.43, 2,312.61)	3.94 (−0.53, 10.18)	2,242.28 (−295.74, 5,721.94)	3.70 (−0.49, 9.49)	152.86 (121.78, 186.29)	−5.93 (−16.73, 5.52)	−0.04 (−0.27, 0.19)
Caribbean	1,021.22 (−134.86, 2,574.50)	4.36 (−0.58, 10.96)	2,368.46 (−317.35, 5,883.10)	4.33 (−0.58, 10.75)	131.92 (115.40, 157.51)	−0.77 (−8.02, 9.36)	−0.02 (−0.13, 0.10)
Central Asia	427.43 (−52.87, 1,145.53)	1.01 (−0.13, 2.72)	912.50 (−114.02, 2,393.25)	1.34 (−0.17, 3.47)	113.49 (97.27, 133.32)	32.64 (22.00, 45.55)	0.93 (0.83, 1.03)
Central Europe	2,406.44 (−304.44, 6,226.35)	1.75 (−0.22, 4.51)	4,505.20 (−591.56, 11,522.33)	1.89 (−0.25, 4.86)	87.21 (73.43, 102.83)	8.13 (0.25, 15.92)	0.23 (−0.03, 0.48)
Central Latin America	3,211.21 (−417.18, 8,165.04)	4.65 (−0.61, 11.73)	10,097.87 (−1,358.49, 25,197.21)	4.29 (−0.58, 10.69)	214.46 (194.28, 237.31)	−7.60 (−13.11, −1.36)	−0.32 (−0.44, −0.20)
Central Sub-Saharan Africa	373.48 (−56.17, 989.09)	2.32 (−0.36, 6.22)	1,109.30 (−171.41, 2,852.09)	2.98 (−0.47, 7.75)	197.02 (147.45, 256.45)	28.43 (5.20, 53.84)	0.84 (0.79, 0.89)
East Asia	17,817.19 (−2,260.87, 46,084.73)	2.91 (−0.38, 7.55)	58,932.91 (−7,972.24, 151,587.17)	3.07 (−0.43, 7.95)	230.76 (182.68, 297.40)	5.58 (−12.67, 26.76)	0.12 (−0.06, 0.30)
Eastern Europe	2,327.60 (−295.56, 6,042.23)	0.91 (−0.12, 2.36)	4,533.17 (−573.61, 11,576.68)	1.25 (−0.16, 3.19)	94.76 (77.87, 115.28)	37.82 (26.07, 51.43)	1.02 (0.81, 1.23)
Eastern Sub-Saharan Africa	1,649.76 (−245.01, 4,297.79)	3.01 (−0.46, 7.79)	3,521.04 (−507.64, 8,956.12)	2.91 (−0.43, 7.46)	113.43 (87.51, 145.66)	−3.63 (−15.53, 11.08)	−0.17 (−0.21, −0.14)
High-income Asia Pacific	2,294.58 (−288.48, 5,951.89)	1.21 (−0.15, 3.11)	5,503.81 (−726.08, 14,140.66)	0.99 (−0.13, 2.54)	139.86 (109.38, 171.13)	−17.73 (−26.40, −10.79)	−0.75 (−0.86, −0.65)
High-income North America	7,211.55 (−914.20, 18,437.23)	1.97 (−0.25, 5.02)	14,693.46 (−1,960.28, 37,550.24)	2.05 (−0.27, 5.23)	103.75 (75.08, 134.23)	3.84 (−10.66, 19.01)	0.22 (0.10, 0.35)
North Africa and Middle East	3,521.77 (−480.60, 8,850.99)	2.78 (−0.39, 6.90)	10,153.42 (−1,335.29, 25,455.06)	2.99 (−0.40, 7.44)	188.30 (144.11, 242.22)	7.45 (−9.34, 28.50)	0.25 (0.22, 0.29)
Oceania	28.88 (−3.88, 75.72)	1.44 (−0.19, 3.77)	78.90 (−10.61, 204.65)	1.50 (−0.21, 3.83)	173.23 (136.39, 210.34)	4.23 (−9.73, 18.26)	0.10 (0.03, 0.17)
South Asia	14,357.86 (−1,979.83, 36,679.76)	3.29 (−0.47, 8.51)	50,411.34 (−7,101.22, 128,758.93)	4.25 (−0.61, 10.80)	251.11 (207.50, 308.76)	29.14 (11.45, 52.51)	0.92 (0.83, 1.01)
Southeast Asia	4,416.95 (−582.42, 11,544.79)	2.14 (−0.29, 5.64)	13,483.76 (−1,738.62, 34,001.99)	2.52 (−0.33, 6.30)	205.27 (172.92, 245.66)	17.86 (3.54, 34.88)	0.44 (0.27, 0.62)
Southern Latin America	448.22 (−55.43, 1,150.95)	1.05 (−0.13, 2.67)	1,028.56 (−136.08, 2,585.40)	1.13 (−0.15, 2.84)	129.48 (104.89, 159.56)	7.97 (−2.50, 19.92)	0.63 (0.39, 0.89)
Southern Sub-Saharan Africa	367.47 (−46.65, 960.24)	1.59 (−0.20, 4.17)	912.27 (−116.41, 2,358.47)	1.99 (−0.26, 5.12)	148.26 (117.49, 184.88)	24.76 (6.54, 44.36)	0.80 (0.51, 1.09)
Tropical Latin America	2,760.56 (−349.35, 7,061.58)	3.71 (−0.48, 9.38)	8,714.54 (−1,110.05, 22,063.12)	3.55 (−0.45, 8.98)	215.68 (194.54, 237.11)	−4.39 (−8.59, 0.32)	−0.20 (−0.33, −0.07)
Western Europe	12,371.60 (−1,646.55, 31,828.26)	2.09 (−0.28, 5.36)	24,605.71 (−3,332.12, 62,513.41)	2.17 (−0.29, 5.60)	98.89 (82.13, 114.99)	4.08 (−2.75, 9.97)	0.18 (−0.02, 0.39)
Western Sub-Saharan Africa	1,475.65 (−208.70, 3,875.79)	2.28 (−0.33, 5.99)	3,605.09 (−506.13, 9,329.30)	2.66 (−0.38, 6.80)	144.31 (114.23, 185.62)	16.67 (0.65, 40.42)	0.36 (0.26, 0.47)

EAPC, estimated annual percentage change; SDI, sociodemographic index; UI, uncertainty interval; DALYs, disability-adjusted life years.

^a^ EAPC is expressed as 95% confidence interval.

^b^ Change shows the percentage change.

### Regional trends by SDI

#### Mortality

**Figure 3:**
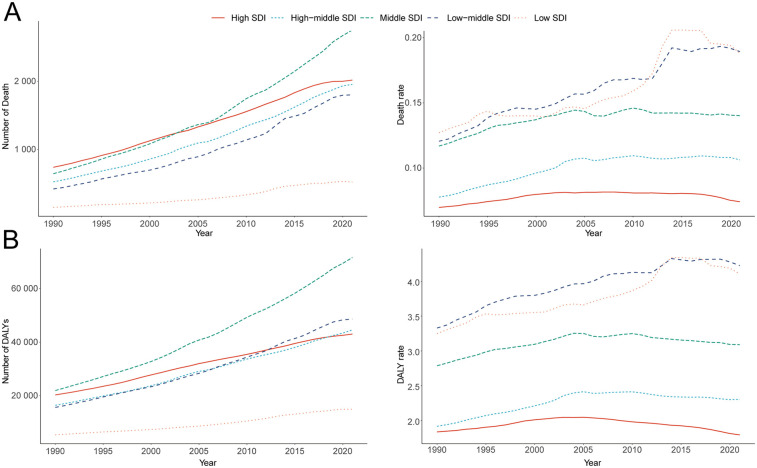
Epidemiologic trends of death, and disability-adjusted life-years (DALYs) rates in 5 sociodemographic index (SDI) regions of atrial fibrillation and flutter due to lead exposure from 1990 to 2021. **(A)** Death casea and rate, **(B)** DALY cases and rate.

Among the five SDI regions, the absolute number of AF/AFL deaths attributable to lead exposure increased markedly. In high SDI areas, deaths rose from 738.25 (95% UI, −106.45 to 1,834.60) in 1990 to 2,017.36 (95% UI, −300.32 to 5,033.64) in 2021 (a 173.26% increase, 95% UI, 148.00–189.93), while in low SDI areas, deaths increased from 150.26 (95% UI, −23.48 to 411.07) to 521.94 (95% UI, −82.57 to 1,375.40), representing a 247.35% increase (95% UI, 185.47–357.45). Notably, the low-middle SDI region experienced an increase in mortality rate from 0.12 (95% UI, −0.02 to 0.32) per 100,000 in 1990 to 0.19 (95% UI, −0.03 to 0.49) in 2021—an increase of 57.06% (95% UI, 29.11–99.88), with an EAPC of 1.54 (95% CI, 1.43–1.64) (Table 1; Figure 3A).

#### DALYs

The greatest increase in DALYs attributable to lead exposure was observed in middle SDI regions, rising from 21,950 (95% UI, −2,769 to 55,497) in 1990 to 71,606 (95% UI, −9,491 to 180,990) in 2021, a 226.23% increase (95% UI, 194.67–268.80). In high SDI regions, DALYs increased from 20,312 (95% UI, −2,620 to 51,855) to 43,025 (95% UI, −5,725 to 110,237). In the low-middle SDI region, DALY rates increased from 3.33 (95% UI, −0.46 to 8.51) in 1990 to 4.23 (95% UI, −0.59 to 10.87) in 2021 (a 26.95% increase, 95% UI, 14.28–45.26), with an EAPC of 0.81 (95% CI, 0.72–0.89) (Table 2; Figure 3B).

### Geographic regional trends

#### Mortality

**Figure 4:**
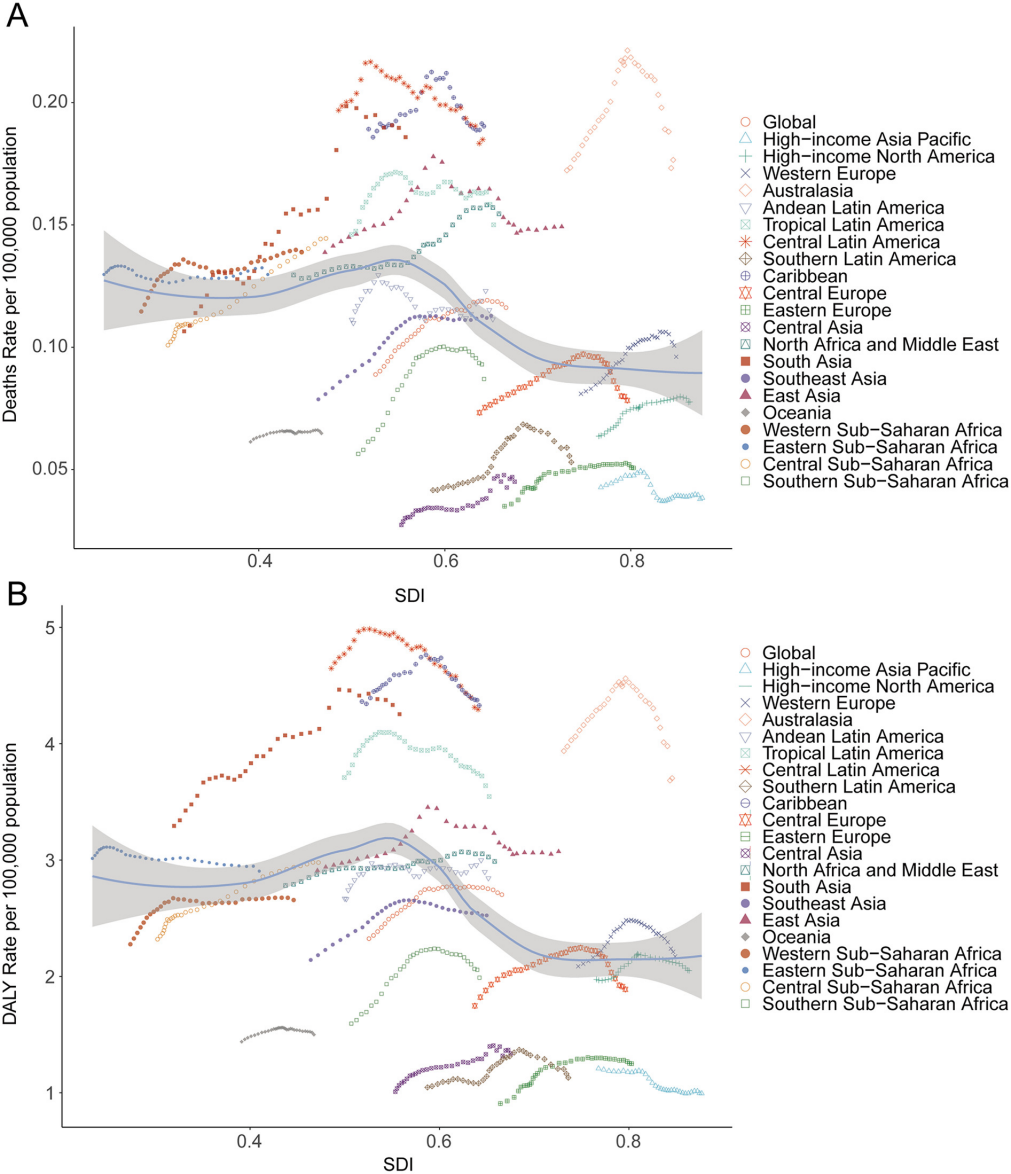
Death and disability-adjusted life-years (DALYs) rates for atrial fibrillation and flutter due to lead exposure from 1990 to 2021. **(A)** Death rate, **(B)** DALY rate.

Among 21 global regions, the greatest absolute increase in lead exposure-related AF/AFL deaths was seen in East Asia, from 523.68 (95% UI, −73.03 to 1,334.10) in 1990 to 2,356.55 (95% UI, −382.39 to 5,904.56) in 2021; Oceania had the lowest absolute deaths (from 0.81 [95% UI, −0.12 to 2.21 in 1990 to 2.51 [95% UI, −0.39 to 6.73 in 2021). In South Asia, the mortality rate rose from 0.11 (95% UI, −0.02 to 0.29) to 0.19 (95% UI, −0.03 to 0.48) during the same period (Table 1). There was a significant negative correlation between SDI and mortality rates from lead-related AF/AFL (Spearman's *r* = −0.29, 95% CI, −0.35 to −0.23; *p* = 2.073 × 10^−15^) (Figure 4A).

#### DALYs

In 2021, East Asia recorded the highest DALYs associated with lead exposure-induced AF/AFL (58,932.91; 95% UI, −7,972.24 to 151,587.17), followed by South Asia (50,411.34; 95% UI, −7,101.22 to 128,758.93), while Oceania reported the lowest burden (78.90; 95% UI, −10.61 to 204.65). High-income North America and Western Europe contributed 14,693.46 (95% UI, −1,960.28 to 37,550.24) and 24,605.71 (95% UI, −3,332.12 to 62,513.41) DALYs, respectively (Table 2). SDI showed a significant negative correlation with DALY burden (*r* = −0.31, 95% CI, −0.38 to −0.25; *p* = 1.520 × 10^−17^) (Figure 4B).

### National trends

#### Mortality

**Figure 5:**
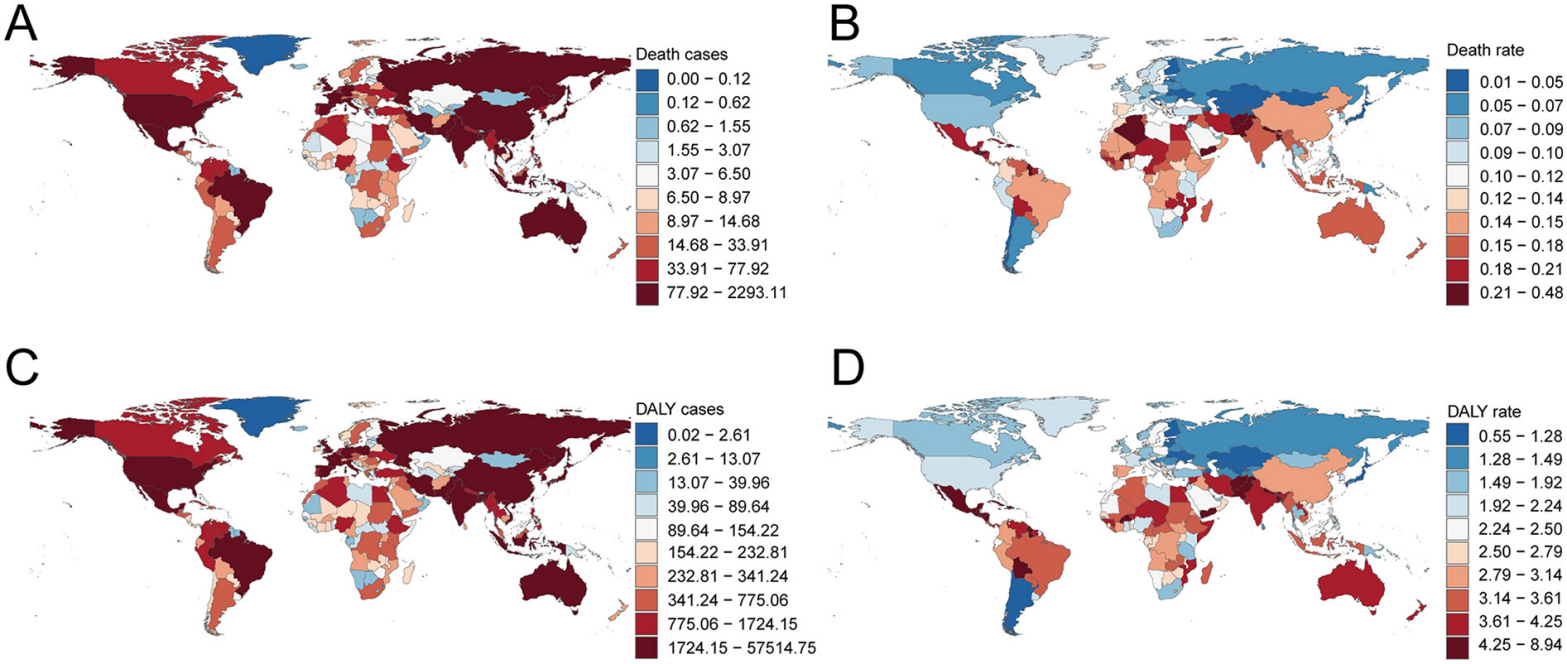
Death and disability-adjusted life-years (DALYs) cases and rates of atrial fibrillation and flutter due to lead exposure in 204 countries and territories. **(A)** Death cases, **(B)** Death rate, **(C)** DALY cases, **(D)** DALY rate. Map created using the rnaturalearth R package, which provides access to Natural Earth map data from https://github.com/ropensci/rnaturalearth.

**Figure 6:**
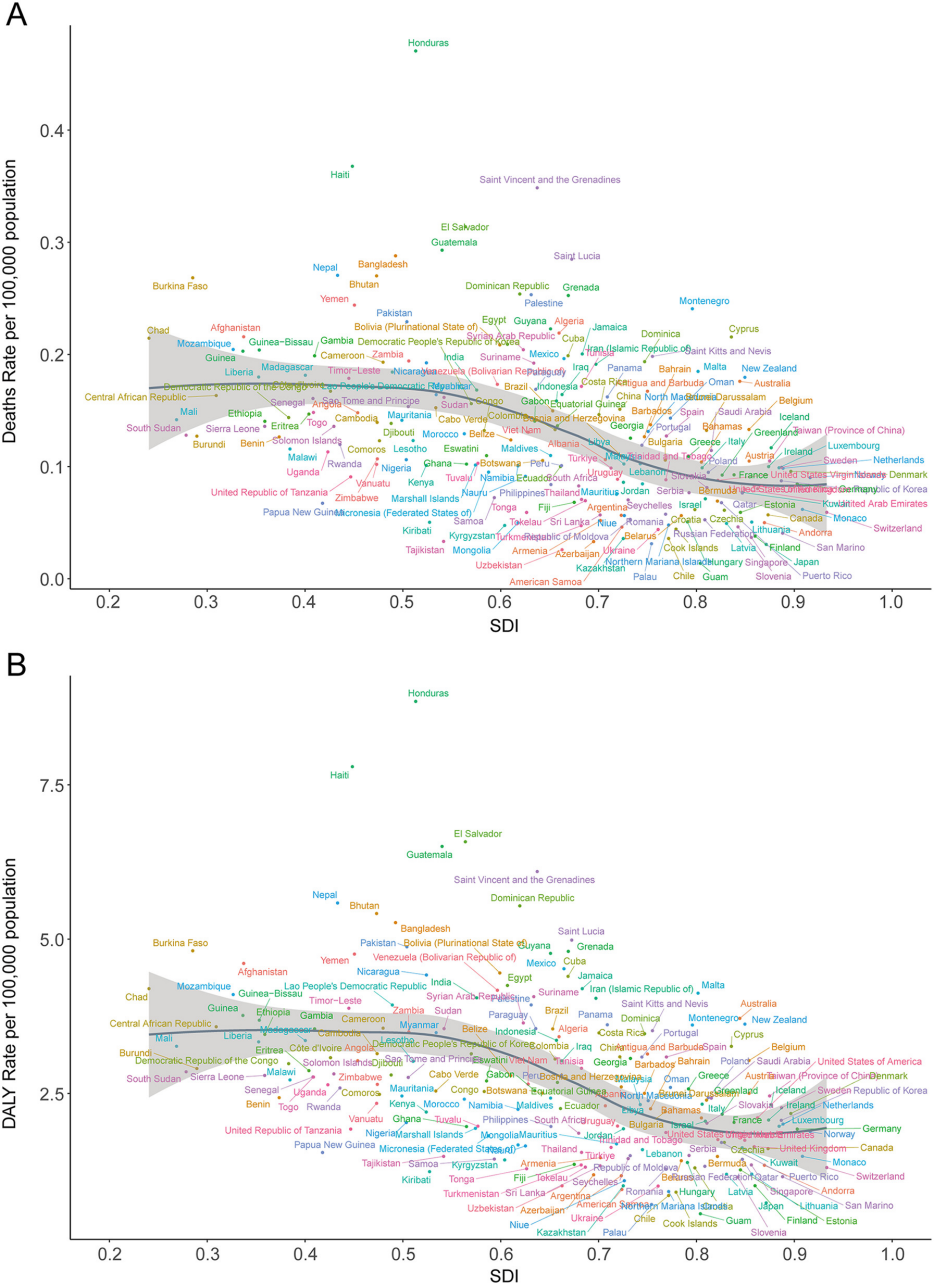
Death and disability-adjusted life-year (DALY) rates of atrial fibrillation and flutter due to lead exposure in 204 countries by SDI in 2021. **(A)** Death rate, **(B)** DALY rate; SDI, socio-demographic index.

In 2021, the top five countries for deaths due to lead exposure-attributable AF/AFL were China (2,270.41; 95% UI, −369.49 to 5,685.05), India (1,352.04; 95% UI, −217.38 to 3,419.69), the United States (543.49; 95% UI, −82.15 to 1,387.91), Brazil (346.66; 95% UI, −52.13 to 867.21), and Germany (222.47; 95% UI, −32.44 to 578.15). The highest mortality rates were observed in Honduras (0.47 per 100,000; 95% UI, −0.07 to 1.20), Haiti (0.37; 95% UI, −0.06 to 0.95), Saint Vincent and the Grenadines (0.35; 95% UI, −0.05 to 0.85), Bangladesh (0.29; 95% UI, −0.05 to 0.75), and Nepal (0.27; 95% UI, −0.04 to 0.71) (Table 1; Supplementary Table S1; Figures 5A,B). Across 204 countries, a strong negative correlation was noted between mortality rate and SDI (Spearman's *r* = −0.53, 95% CI, −0.60 to −0.42; *p* < 0.001) (Figure 6A).

#### DALYs

In 2021, the highest national DALY counts were in the United States (13,403.82; 95% UI, −1,800.56 to 34,335.95), India (39,509.42; 95% UI, −5,576.83 to 100,784.23), and China (56,945.30; 95% UI, −7,718.89 to 146,211.01). The lowest DALY burdens were reported in small Pacific Island nations, such as Kiribati (0.60; 95% UI, −0.08 to 1.58) and Nauru (0.06; 95% UI, −0.01 to 0.16). The highest DALY rates per 100,000 population were seen in Honduras (8.85; 95% UI, −1.27 to 21.78), Haiti (7.79; 95% UI, −1.10 to 19.87), Guatemala (6.50; 95% UI, −0.89 to 16.46), El Salvador (6.58; 95% UI, −0.87 to 16.56), and Saint Vincent and the Grenadines (6.10; 95% UI, −0.82 to 15.43) (Table 2; Supplementary Table S2; Figures 5C,D). Again, there was a significant negative correlation between national DALY rates and SDI (Spearman's *r* = −0.53, 95% CI, −0.60 to −0.43; *p* < 0.001) (Figure 6B).

### Age, period and cohort effects of AF/AFL deaths due to lead exposure

**Figure 7:**
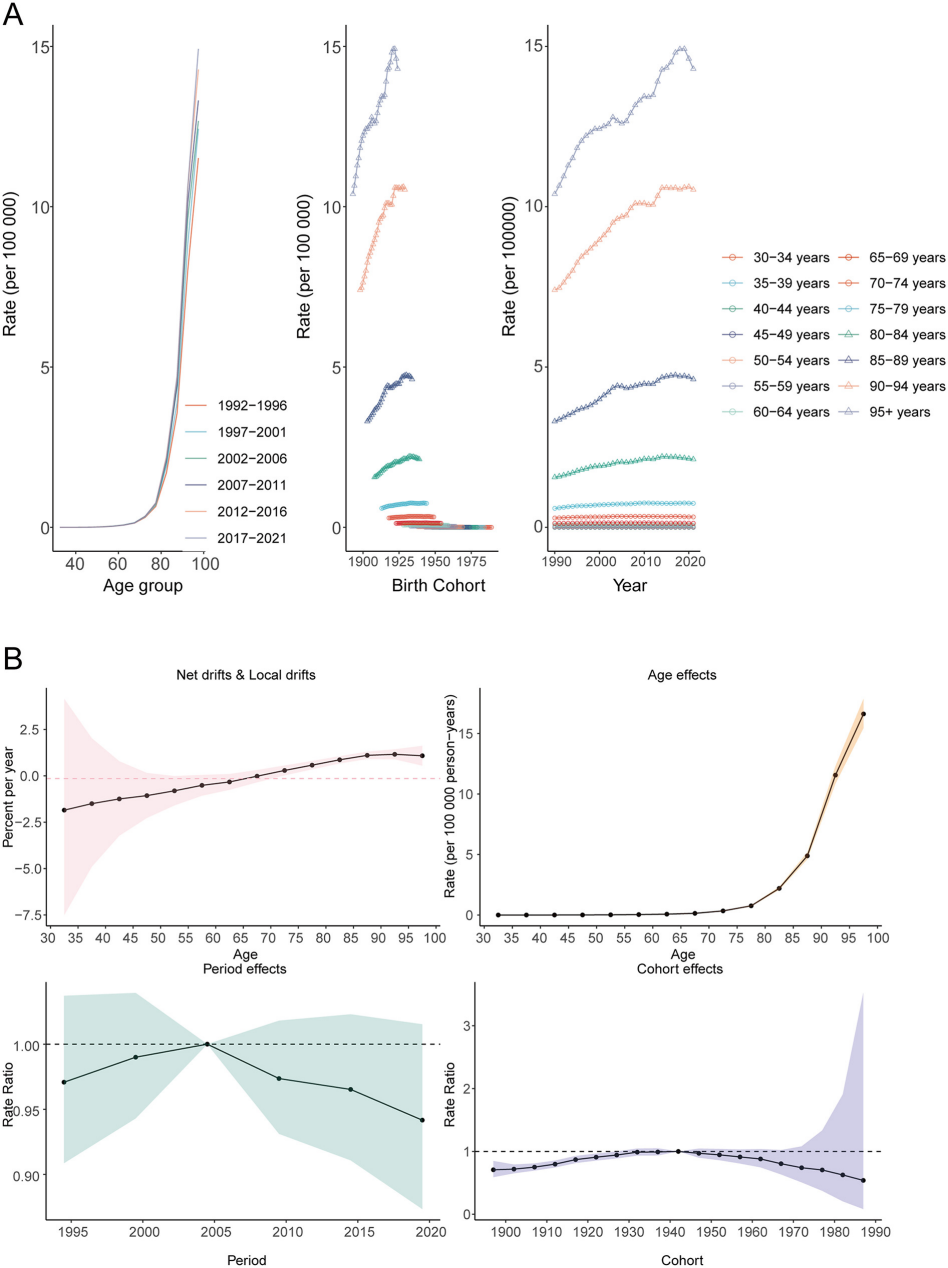
Age, period, and cohort effects on atrial fibrillation and flutter mortality due to lead exposure globally. **(A)** Age-specific rates by age, **(B)** Net-drift.

From 1992 to 2021, age-specific mortality rates for AF/AFL attributable to lead exposure demonstrated a clear age-dependent increase. Mortality rates rose exponentially with age, being lowest in the 30–34 years group (0.00034 in 1992–1996) and highest among those aged ≥95 years (14.92 in 2017–2021). Mortality increased sharply after age 75 (e.g., for the 75–79 age group, from 0.65 in 1992–1996 to 0.76 in 2017–2021). For the same birth cohort, mortality rates increased markedly with age (e.g., for those born in 1958, mortality was 0.00034 at ages 30–34, rising to 0.068 at ages 60–64). Local drift analysis showed negative average annual changes in mortality for younger age groups (−1.847 to −0.327 for ages 32.5–62.5 years) and positive changes for older age groups (0.291–1.161 for ages 72.5–97.5 years). Net drift analysis indicated no significant overall trend in mortality (average annual change −0.146; 95% CI, −0.614 to 0.325), consistent with the findings from the period effect analysis (Figures 7A,B).

### BAPC prediction of the death rate and DALY rate

**Figure 8:**
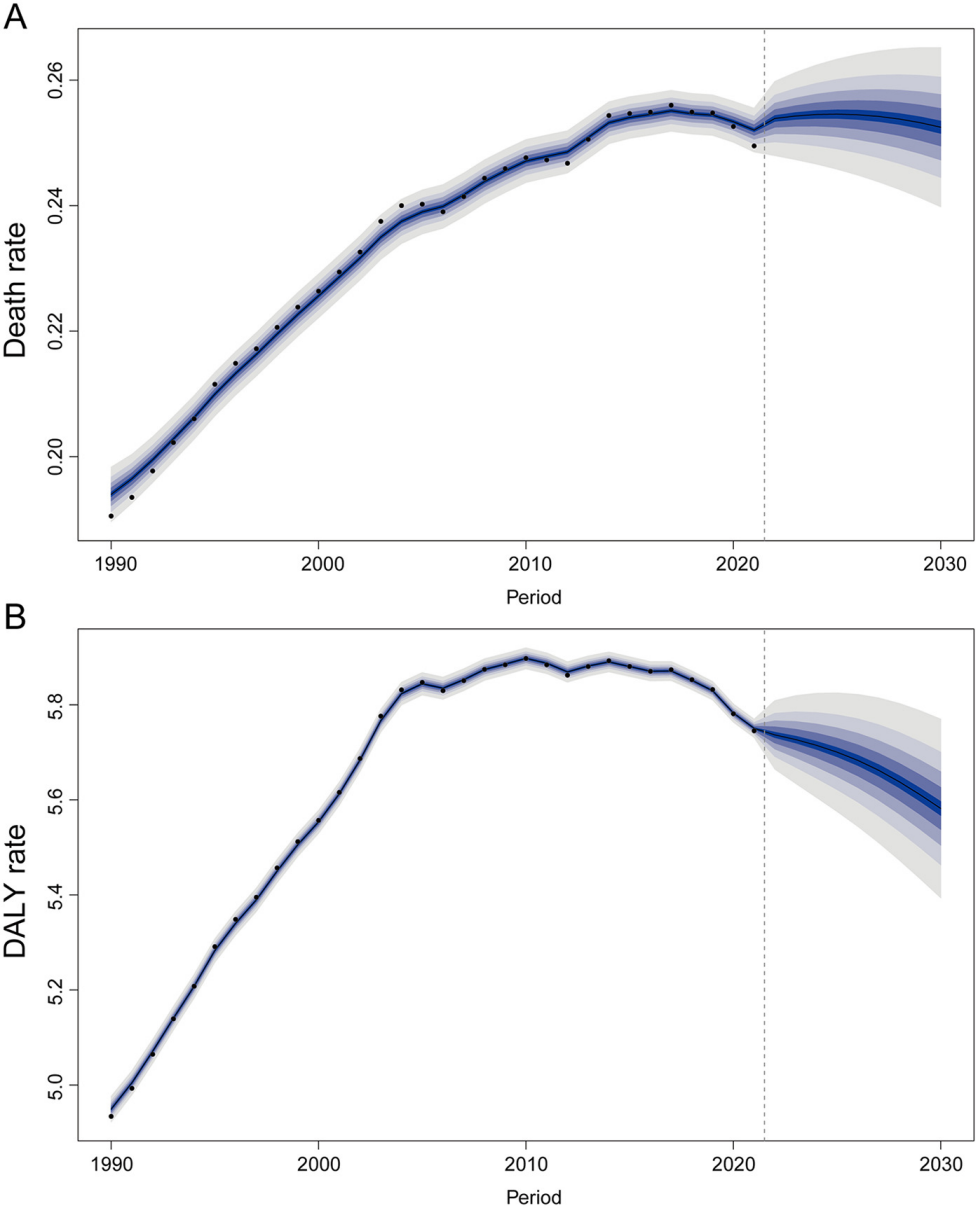
The trends of death and disability-adjusted life-year (DALY) rates for atrial fibrillation and flutter due to lead exposure globally from 2022 to 2030 predicted by the Bayesian age-period-cohort model. **(A)** Death rate, **(B)** DALY rate.

From 2022 to 2030, a slight increase in AF/AFL-related mortality and DALY rate attributable to lead exposure is projected (Figures 8A,B). By 2030, the estimated number of AF/AFL-related deaths caused by lead exposure is approximately 11,880, with DALY cases reaching around 262,634 (Supplementary Figure S1).

## Discussion

In this global analysis of GBD 2021 data, we found that AF/AFL attributable to lead exposure remains a significant and rising public health burden worldwide. **First**, the overall AF/AFL burden associated with lead exposure has increased substantially over the past three decades, in parallel with the growing global incidence of AF/AFL (25). In 2019, an estimated tens of thousands of AF/AFL cases, and a notable fraction of AF-related DALYs and deaths, were attributable to lead exposure, underlining lead as an important modifiable risk contributor to AF/AFL at the global level (26). Second, our age-specific analyses highlight older adults as the most affected group: lead-related AF/AFL burden was concentrated in older age cohorts, with a steep rise in attributable AF/AFL mortality and DALYs beyond the age of 60. This reflects the well-known epidemiology of AF as an age-dependent condition—the majority of patients are diagnosed at ≥60 years, and incidence increases sharply with age (27, 28), combined with the cumulative nature of lead exposure over the life course. Third, we identified pronounced disparities by socio-demographic development. Regions with low Socio-Demographic Index (SDI) experienced disproportionately higher AF/AFL burden due to lead, whereas high-SDI regions had much lower attributable rates. In low and middle-income countries, both historical and ongoing lead exposures (e.g., through leaded fuels, industrial emissions, informal battery recycling, contaminated consumer products) have sustained higher population lead burdens, which translate into greater AF/AFL risk (29). Finally, we observed notable sex-specific patterns. Overall, males showed higher AF/AFL mortality and DALY rates attributable to lead than females in 2021, consistent with historically higher lead exposures in men (e.g., occupational exposures) and higher baseline AF risk in males (30). However, the sex gap appears to be narrowing as population aging and improved AF detection in women have increased the absolute number of AF cases in women (especially in high-income countries) (31). These key findings underscore that lead exposure contributes appreciably to the global AF/AFL epidemic, particularly among vulnerable demographics (older adults, males) and in less-developed regions.

Our findings suggest that lead exposure is an under-recognized yet important contributor to AF/AFL burden, acting through multiple direct and indirect mechanisms. Biologically, chronic lead exposure induces cardiovascular changes that plausibly promote atrial arrhythmogenesis. Lead is a known potent inducer of oxidative stress and inflammation: it impairs glutathione and antioxidant enzyme activity, generates excess reactive oxygen species, and disrupts nitric oxide signaling, leading to endothelial dysfunction and a pro-inflammatory state (32, 33). These processes contribute to atrial structural remodeling (e.g., fibrosis) and electrical disturbances that favor the development of AF. Experimental evidence supports direct electrophysiological effects of lead on the myocardium. Lead ions can substitute for calcium and block L-type calcium channels (Cav1.2) in cardiomyocytes (34). Acute cardiac exposure to lead has been shown to reduce the cardiac action potential plateau, precipitate early after-depolarizations, and increase arrhythmia susceptibility in animal models (34).

Importantly, many of lead's cardiovascular effects are **indirect**, acting via established AF risk factors. Chronic lead exposure contributes to hypertension, coronary artery disease, and left ventricular dysfunction (35, 36). Lead-induced oxidative stress and inflammation promote atherosclerosis and arterial stiffness, while lead's competition with calcium can trigger neurohormonal changes that elevate blood pressure (33). Given that hypertension, ischemic heart disease, and heart failure are major risk factors for AF, lead exposure likely increases AF risk in part by amplifying these intermediate conditions (37). In summary, chronic lead exposure likely increases AF/AFL risk through a multifactorial mechanism: direct electrophysiological perturbation of atrial tissue and indirect promotion of AF risk factors (notably hypertension and atherosclerotic cardiovascular disease).

Notably, there has been scarce direct epidemiological research focusing on lead and atrial arrhythmias before our study. The absence of extensive prior literature on lead-induced AF may be due to under-recognition; AF onset is multifactorial and often occurs decades after lead exposure, complicating attribution. Nevertheless, our results align with the limited studies that hint at such a link. For example, an analysis of the Normative Aging Study cohort found that cumulative lead dose (measured via bone lead) was associated with prolonged cardiac conduction intervals and a higher likelihood of atrial and ventricular conduction blocks on ECG (38). Although that study did not specifically track AF incidence, it demonstrated that lifelong lead accumulation can produce electrophysiological changes, such as QT prolongation, that predispose to arrhythmia. Navas-Acien et al. reviewed evidence on lead and cardiovascular disease and noted biologically plausible links to arrhythmic outcomes, though at the time, data on AF were virtually non-existent (39). More recently, the broader concept of the “environmental exposome” in cardiovascular disease has gained traction. A 2024 Circulation Research review underscored that environmental toxicants—including heavy metals like lead—may be important, if under-appreciated, contributors to the rising global AF burden (40). Our study provides concrete epidemiological evidence to support this notion, quantifying AF risk from an environmental pollutant at a global scale. In comparison with traditional AF risk factors, lead's impact remains smaller than that of hypertension or obesity in high-SDI countries. However, in certain low-SDI regions or subpopulations with high lead exposures, the population-attributable fraction of AF due to lead is likely much higher than global averages (41). Overall, our results are in agreement with and add nuance to the growing literature that elevated lead exposure in populations contributes to the epidemiology of AF/AFL, particularly via its effect on upstream conditions. We also underscore, for the first time, the future trajectory of this problem by forecasting trends—an area where comparative data are lacking. While previous GBD analyses noted relatively stagnant age-standardized AF incidence globally from 1990 to 2019 (26), our finding of an increasing lead-attributable AF burden in low-SDI regions suggests that, without intervention, environmental lead could fuel a continued rise in AF in vulnerable regions, even if AF rates plateau in high-SDI countries.

Our analysis has several important public health implications. It reveals that a substantial portion of the world's AF/AFL burden is theoretically preventable by eliminating lead exposure, a modifiable environmental risk factor. Unlike non-modifiable AF risk factors such as aging or genetic predisposition, lead exposure can be reduced through policy and regulatory action. The fact that lead contributes to AF/AFL in addition to its known effects on IQ, chronic kidney disease, and other cardiovascular endpoints means interventions against lead will yield multifaceted health benefits. Below, we outline key public health and policy measures suggested by our findings:

1. Strengthen Global Lead Surveillance and Regulation: There is an urgent need for improved monitoring of population lead exposure and identification of exposure sources, especially in low- and middle-income countries.
2. Mitigate Major Ongoing Sources of Lead: Policy measures should focus on eliminating or controlling the dominant sources of lead exposure in high-burden regions. These include informal lead-acid battery recycling operations, lead contaminants in spices/foodstuffs, lead-based paints and ceramics, cookware made from lead-containing alloys, and residual leaded gasoline emissions in certain areas.
3. Integrate Environmental Risk into Cardiovascular Prevention: Clinicians and public health authorities should recognize lead exposure as a modifiable cardiovascular risk factor, akin to smoking or air pollution, and incorporate it into AF/AFL prevention strategies.

By implementing these measures, countries can expect meaningful reductions in AF incidence over time, along with broad health and economic gains. Notably, the economic cost of lead exposure is enormous, so the cost-benefit ratio of prevention is highly favorable. Our findings reinforce that investing in lead pollution control is an investment in cardiovascular health and healthy aging worldwide.

In summary, while our study leverages the best available global data and standardized methods to quantify lead's contribution to AF, these limitations highlight the need for cautious interpretation. Further research—including longitudinal cohort studies in exposed populations and improved surveillance in low-SDI regions—is needed to validate the causal links and refine these estimates. Notwithstanding these caveats, our analysis provides a valuable big-picture assessment that highlights an actionable environmental determinant of AF on a worldwide scale.

### Limitation

This study had some important limitations. First, Our analysis considered AF and atrial flutter together, and did not differentiate between paroxysmal, persistent, or chronic AF. GBD case definitions rely on clinical and epidemiological data that may vary in accuracy. It is unclear if lead exposure might be more strongly associated with certain subtypes of AF (e.g., permanent AF in older patients with comorbidities) than others. Second, High-quality data on both AF/AFL epidemiology and population lead exposure are lacking in some regions. Many low-SDI countries have scarce surveillance for AF (which can be asymptomatic and underdiagnosed) and have not conducted recent population-based lead surveys.

## Conclusion

This study provides the first comprehensive global assessment of atrial fibrillation and flutter attributable to lead exposure. Our results demonstrate a substantial and growing burden, especially in older populations, males, and low-SDI regions. These findings highlight environmental lead as an under-recognized but modifiable risk factor for AF/AFL worldwide. Targeted public health interventions and regulatory policies to further reduce lead exposure are urgently needed and are likely to yield significant reductions in the global burden of AF/AFL, along with broader cardiovascular and societal benefits.

## Data Availability

The original contributions presented in the study are included in the article/Supplementary Material, further inquiries can be directed to the corresponding authors.
