# Supplementary figures and images for: The burden and trend prediction of atrial fibrillation and flutter associated with lead exposure: insights from the global burden of disease study 2021
Source: Front Cardiovasc Med. 2025 Aug 12;12:1638747. doi: 10.3389/fcvm.2025.1638747 (PMC12378705; doi:10.3389/fcvm.2025.1638747)

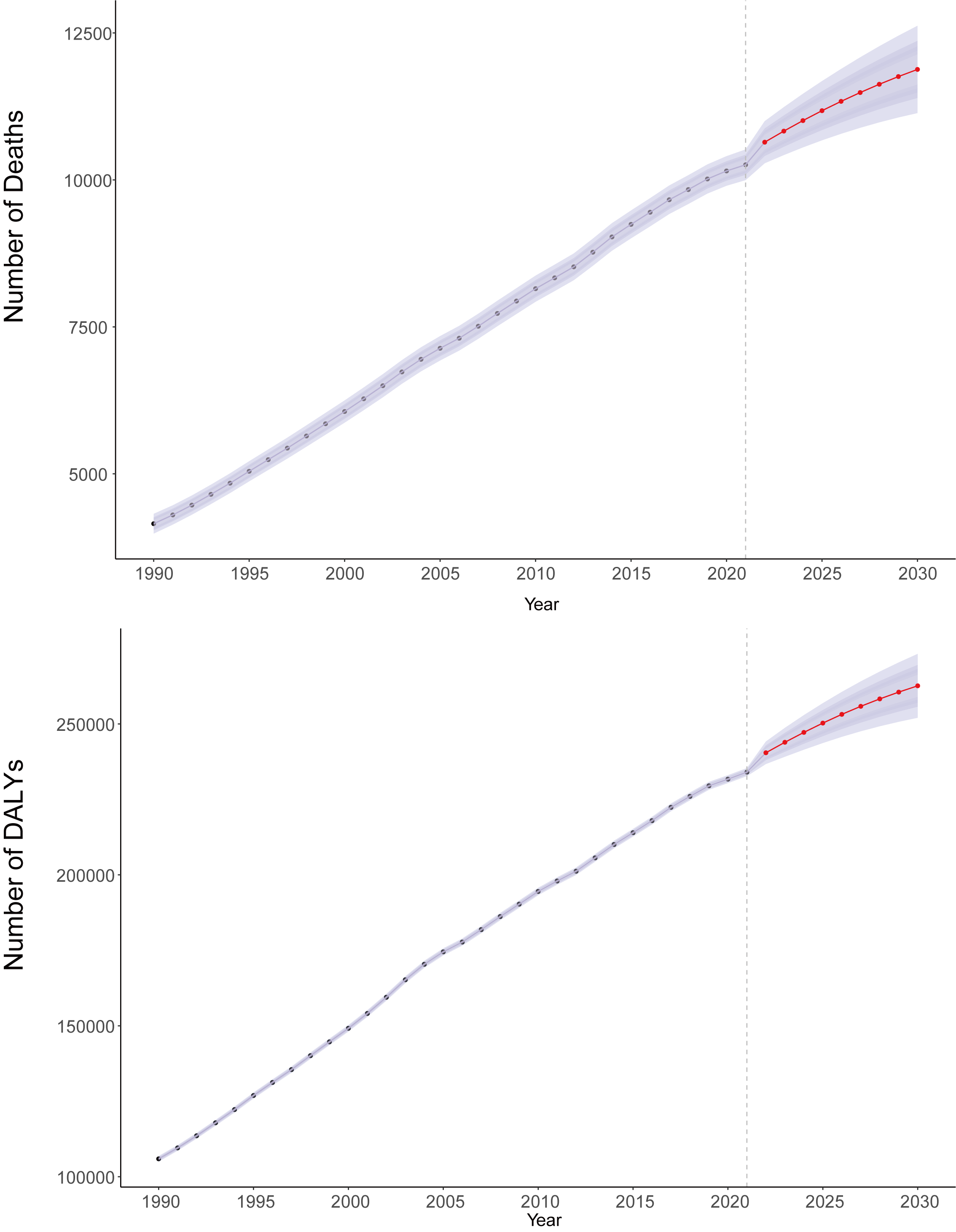

Supplement: Supplementary file 1 [file Image1.tif]
