# Supplementary material for: The burden and trend prediction of atrial fibrillation and flutter associated with lead exposure: insights from the global burden of disease study 2021
Source: Front Cardiovasc Med. 2025 Aug 12;12:1638747. doi: 10.3389/fcvm.2025.1638747 (PMC12378705; doi:10.3389/fcvm.2025.1638747)
Supplement: Supplementary file 2 [file Table1.docx]

Table S1: Mortality of lead exposure-related atrial fibrillation and flutter between 1990 and 2021 at the national level

| location | 1990 | |  | 2021 | |  | 1990-2021 | |
| --- | --- | --- | --- | --- | --- | --- | --- | --- |
|  | Death cases | Death rate |  | Death cases | Death rates |  | Rate change | EAPC |
| Afghanistan | 6.39(-1.01,18.60) | 0.16(-0.02,0.46) |  | 11.44(-1.86,30.98) | 0.22(-0.04,0.59) |  | 38.51(7.56,94.48) | 1.13(1.06,1.21) |
| Albania | 1.09(-0.17,2.82) | 0.07(-0.01,0.19) |  | 4.04(-0.67,10.34) | 0.11(-0.02,0.27) |  | 45.94(4.63,105.30) | 1.55(1.33,1.78) |
| Algeria | 8.14(-1.20,21.08) | 0.18(-0.03,0.48) |  | 38.98(-5.20,97.72) | 0.22(-0.03,0.55) |  | 22.80(-9.72,75.69) | 1.41(1.12,1.70) |
| American Samoa | 0.00(-0.00,0.01) | 0.04(-0.01,0.11) |  | 0.01(-0.00,0.04) | 0.05(-0.01,0.12) |  | 7.26(-16.69,34.69) | 0.38(0.33,0.43) |
| Andorra | 0.02(-0.00,0.05) | 0.05(-0.01,0.14) |  | 0.10(-0.01,0.25) | 0.05(-0.01,0.13) |  | -4.44(-39.79,39.72) | 0.10(-0.16,0.37) |
| Angola | 2.05(-0.31,5.31) | 0.10(-0.02,0.29) |  | 8.22(-1.43,21.41) | 0.15(-0.03,0.38) |  | 42.95(-1.17,106.38) | 1.06(1.01,1.11) |
| Antigua and Barbuda | 0.09(-0.01,0.24) | 0.16(-0.02,0.41) |  | 0.13(-0.02,0.32) | 0.17(-0.02,0.42) |  | 5.16(-10.02,21.50) | 0.30(-0.00,0.60) |
| Argentina | 11.54(-1.57,29.39) | 0.04(-0.01,0.11) |  | 33.57(-4.89,84.42) | 0.06(-0.01,0.14) |  | 26.60(12.03,41.05) | 1.51(1.13,1.89) |
| Armenia | 0.51(-0.07,1.36) | 0.03(-0.00,0.07) |  | 2.21(-0.31,5.61) | 0.05(-0.01,0.13) |  | 104.79(61.57,154.18) | 2.72(2.32,3.12) |
| Australia | 29.75(-4.39,74.52) | 0.17(-0.03,0.44) |  | 99.76(-14.50,250.36) | 0.18(-0.03,0.45) |  | 0.59(-11.93,14.77) | 0.24(-0.11,0.59) |
| Austria | 9.50(-1.41,23.73) | 0.08(-0.01,0.20) |  | 26.02(-3.93,68.22) | 0.10(-0.02,0.28) |  | 30.88(9.13,53.71) | 0.95(0.52,1.39) |
| Azerbaijan | 0.94(-0.14,2.45) | 0.03(-0.00,0.07) |  | 2.22(-0.34,5.73) | 0.03(-0.01,0.08) |  | 24.91(-12.37,70.78) | 1.25(0.99,1.52) |
| Bahamas | 0.10(-0.02,0.26) | 0.09(-0.01,0.22) |  | 0.31(-0.05,0.81) | 0.10(-0.02,0.27) |  | 20.17(-4.70,49.32) | 0.43(0.08,0.79) |
| Bahrain | 0.08(-0.01,0.24) | 0.15(-0.02,0.45) |  | 0.34(-0.05,0.94) | 0.14(-0.02,0.39) |  | -10.69(-35.62,16.94) | -0.46(-0.99,0.07) |
| Bangladesh | 54.27(-7.99,154.52) | 0.17(-0.03,0.49) |  | 254.53(-40.96,662.34) | 0.29(-0.05,0.75) |  | 66.52(24.75,167.98) | 1.42(0.94,1.90) |
| Barbados | 0.35(-0.05,0.88) | 0.12(-0.02,0.30) |  | 0.65(-0.09,1.62) | 0.13(-0.02,0.32) |  | 5.30(-13.71,27.72) | 0.15(-0.08,0.38) |
| Belarus | 4.39(-0.67,11.94) | 0.04(-0.01,0.10) |  | 9.40(-1.41,23.75) | 0.06(-0.01,0.14) |  | 47.03(15.63,95.02) | 1.09(0.94,1.24) |
| Belgium | 20.75(-2.96,53.56) | 0.14(-0.02,0.35) |  | 44.68(-7.28,110.94) | 0.13(-0.02,0.33) |  | -2.11(-16.30,13.83) | 0.32(-0.04,0.69) |
| Belize | 0.10(-0.02,0.26) | 0.11(-0.02,0.28) |  | 0.29(-0.04,0.72) | 0.12(-0.02,0.31) |  | 11.10(-14.61,43.00) | 0.12(-0.44,0.68) |
| Benin | 1.30(-0.19,3.38) | 0.09(-0.01,0.24) |  | 3.69(-0.57,9.62) | 0.13(-0.02,0.33) |  | 34.54(1.13,84.70) | 0.97(0.85,1.09) |
| Bermuda | 0.05(-0.01,0.12) | 0.10(-0.01,0.25) |  | 0.12(-0.02,0.29) | 0.07(-0.01,0.17) |  | -28.54(-41.93,-5.86) | -1.28(-1.42,-1.15) |
| Bhutan | 0.18(-0.03,0.49) | 0.15(-0.02,0.41) |  | 1.30(-0.21,3.21) | 0.27(-0.04,0.68) |  | 80.91(21.57,168.69) | 2.09(2.03,2.16) |
| Bolivia (Plurinational State of) | 3.44(-0.50,9.20) | 0.17(-0.02,0.44) |  | 13.14(-1.89,35.96) | 0.21(-0.03,0.56) |  | 26.13(-2.60,72.69) | 0.86(0.77,0.96) |
| Bosnia and Herzegovina | 2.26(-0.32,5.91) | 0.08(-0.01,0.21) |  | 6.94(-0.97,17.54) | 0.11(-0.02,0.28) |  | 40.47(3.50,99.04) | 1.16(1.02,1.29) |
| Botswana | 0.30(-0.05,0.77) | 0.11(-0.02,0.29) |  | 0.84(-0.12,2.18) | 0.11(-0.01,0.27) |  | -6.32(-37.38,41.19) | 0.01(-0.20,0.23) |
| Brazil | 78.23(-11.51,192.18) | 0.15(-0.02,0.36) |  | 346.66(-52.13,867.21) | 0.15(-0.02,0.37) |  | 1.72(-4.91,7.67) | 0.03(-0.14,0.19) |
| Brunei Darussalam | 0.11(-0.02,0.29) | 0.15(-0.02,0.40) |  | 0.23(-0.03,0.60) | 0.13(-0.02,0.34) |  | -14.37(-35.93,19.79) | 0.21(-0.06,0.49) |
| Bulgaria | 5.91(-0.86,15.87) | 0.08(-0.01,0.23) |  | 15.43(-2.10,38.51) | 0.11(-0.01,0.26) |  | 28.51(3.09,61.12) | 0.94(0.77,1.11) |
| Burkina Faso | 3.63(-0.54,9.47) | 0.17(-0.03,0.42) |  | 14.05(-2.42,37.51) | 0.27(-0.05,0.72) |  | 62.08(21.04,127.72) | 1.88(1.70,2.07) |
| Burundi | 1.83(-0.24,5.24) | 0.13(-0.02,0.35) |  | 3.07(-0.42,8.99) | 0.13(-0.02,0.36) |  | 0.71(-31.07,46.92) | -0.34(-0.52,-0.15) |
| Cabo Verde | 0.22(-0.03,0.56) | 0.09(-0.01,0.24) |  | 0.63(-0.09,1.67) | 0.15(-0.02,0.40) |  | 62.66(-5.06,169.13) | 1.37(1.14,1.60) |
| Cambodia | 1.88(-0.29,5.33) | 0.08(-0.01,0.23) |  | 8.97(-1.26,24.02) | 0.14(-0.02,0.39) |  | 74.66(28.05,130.38) | 1.83(1.76,1.91) |
| Cameroon | 3.61(-0.53,9.65) | 0.15(-0.02,0.41) |  | 11.95(-1.75,31.10) | 0.19(-0.03,0.50) |  | 26.37(-7.32,95.87) | 0.55(0.45,0.66) |
| Canada | 18.27(-2.63,45.65) | 0.06(-0.01,0.15) |  | 50.31(-7.34,127.79) | 0.06(-0.01,0.14) |  | -3.95(-14.78,6.99) | -0.33(-0.48,-0.18) |
| Central African Republic | 0.73(-0.12,1.96) | 0.13(-0.02,0.35) |  | 1.55(-0.24,4.33) | 0.16(-0.03,0.48) |  | 28.46(-3.50,66.29) | 0.84(0.76,0.93) |
| Chad | 2.32(-0.37,6.35) | 0.13(-0.02,0.35) |  | 6.24(-0.94,16.09) | 0.21(-0.03,0.55) |  | 67.21(23.45,140.54) | 1.65(1.49,1.81) |
| Chile | 2.14(-0.31,5.50) | 0.03(-0.00,0.07) |  | 9.57(-1.44,23.83) | 0.04(-0.01,0.09) |  | 31.16(11.06,55.33) | 2.02(1.45,2.60) |
| China | 503.23(-70.02,1282.18) | 0.14(-0.02,0.36) |  | 2270.41(-369.49,5685.05) | 0.15(-0.02,0.38) |  | 8.13(-22.12,48.89) | 0.10(-0.16,0.36) |
| Colombia | 20.75(-3.13,52.12) | 0.17(-0.03,0.42) |  | 77.92(-10.88,195.53) | 0.13(-0.02,0.33) |  | -21.04(-35.02,-5.43) | -1.12(-1.27,-0.97) |
| Comoros | 0.15(-0.02,0.41) | 0.14(-0.02,0.39) |  | 0.37(-0.06,0.98) | 0.12(-0.02,0.33) |  | -13.54(-40.41,21.83) | -0.65(-0.73,-0.56) |
| Congo | 0.67(-0.11,1.70) | 0.12(-0.02,0.31) |  | 1.69(-0.28,4.40) | 0.13(-0.02,0.35) |  | 11.00(-19.42,50.12) | 0.23(0.14,0.32) |
| Cook Islands | 0.00(-0.00,0.01) | 0.06(-0.01,0.15) |  | 0.01(-0.00,0.03) | 0.04(-0.01,0.12) |  | -26.33(-44.88,-4.90) | -0.99(-1.02,-0.96) |
| Costa Rica | 2.10(-0.34,5.32) | 0.14(-0.02,0.35) |  | 8.60(-1.34,21.40) | 0.15(-0.02,0.36) |  | 5.62(-12.23,28.98) | 0.02(-0.20,0.24) |
| Croatia | 2.01(-0.30,5.15) | 0.04(-0.01,0.11) |  | 6.38(-0.96,16.19) | 0.06(-0.01,0.16) |  | 45.14(16.92,74.13) | 0.72(0.58,0.85) |
| Cuba | 15.27(-2.25,38.34) | 0.18(-0.03,0.45) |  | 44.57(-6.72,109.59) | 0.20(-0.03,0.49) |  | 11.95(-5.99,33.10) | 1.33(1.01,1.66) |
| Cyprus | 1.45(-0.21,4.07) | 0.34(-0.05,0.94) |  | 3.19(-0.48,7.98) | 0.22(-0.03,0.55) |  | -37.25(-55.86,-5.88) | 0.37(0.25,0.49) |
| Czechia | 5.85(-0.82,15.03) | 0.05(-0.01,0.12) |  | 14.68(-2.09,37.25) | 0.06(-0.01,0.15) |  | 28.46(11.00,49.33) | -1.62(-1.82,-1.41) |
| C么te d'Ivoire | 2.02(-0.32,5.28) | 0.13(-0.02,0.33) |  | 8.52(-1.25,20.86) | 0.17(-0.02,0.42) |  | 29.36(-6.79,75.08) | 1.00(0.83,1.17) |
| Democratic People's Republic of Korea | 10.32(-1.47,27.93) | 0.12(-0.02,0.33) |  | 35.77(-5.66,95.90) | 0.16(-0.02,0.42) |  | 28.76(-2.99,77.09) | 1.12(0.93,1.31) |
| Democratic Republic of the Congo | 7.06(-1.00,19.84) | 0.10(-0.01,0.27) |  | 26.88(-3.97,75.04) | 0.14(-0.02,0.40) |  | 50.57(5.83,112.90) | 1.41(1.24,1.58) |
| Denmark | 5.26(-0.76,13.26) | 0.06(-0.01,0.15) |  | 13.84(-1.97,34.83) | 0.10(-0.01,0.24) |  | 59.03(35.06,84.18) | 1.56(0.93,2.18) |
| Djibouti | 0.08(-0.01,0.22) | 0.14(-0.02,0.37) |  | 0.35(-0.05,0.88) | 0.14(-0.02,0.35) |  | -3.31(-29.92,34.01) | -0.24(-0.29,-0.19) |
| Dominica | 0.10(-0.01,0.25) | 0.19(-0.03,0.48) |  | 0.13(-0.02,0.34) | 0.19(-0.03,0.50) |  | 2.71(-17.45,31.03) | -0.00(-0.19,0.18) |
| Dominican Republic | 6.54(-0.98,16.35) | 0.29(-0.04,0.73) |  | 23.71(-3.86,58.53) | 0.25(-0.04,0.63) |  | -13.10(-34.85,17.89) | -0.08(-0.45,0.28) |
| Ecuador | 4.27(-0.62,11.05) | 0.11(-0.02,0.29) |  | 13.67(-2.07,33.98) | 0.10(-0.02,0.25) |  | -10.37(-26.01,10.92) | -0.29(-0.49,-0.09) |
| Egypt | 23.34(-3.75,58.81) | 0.21(-0.03,0.53) |  | 53.77(-7.98,131.74) | 0.21(-0.03,0.50) |  | 1.60(-24.91,42.34) | 0.23(0.10,0.35) |
| El Salvador | 7.57(-1.16,19.48) | 0.27(-0.04,0.70) |  | 23.82(-3.43,58.54) | 0.31(-0.04,0.76) |  | 14.82(-12.59,52.76) | 0.38(0.26,0.50) |
| Equatorial Guinea | 0.12(-0.02,0.31) | 0.11(-0.02,0.28) |  | 0.36(-0.06,0.93) | 0.14(-0.02,0.34) |  | 28.74(-22.69,119.39) | 0.80(0.75,0.86) |
| Eritrea | 0.57(-0.08,1.59) | 0.12(-0.02,0.35) |  | 1.66(-0.22,4.63) | 0.15(-0.02,0.40) |  | 20.41(-24.23,115.29) | 0.43(0.31,0.55) |
| Estonia | 0.59(-0.08,1.49) | 0.03(-0.00,0.08) |  | 2.11(-0.32,5.35) | 0.06(-0.01,0.15) |  | 80.41(49.79,113.65) | 1.75(1.66,1.85) |
| Eswatini | 0.18(-0.03,0.48) | 0.11(-0.02,0.30) |  | 0.32(-0.05,0.85) | 0.11(-0.02,0.29) |  | -1.28(-32.73,43.07) | 0.84(0.38,1.29) |
| Ethiopia | 12.60(-1.72,37.12) | 0.13(-0.02,0.39) |  | 37.06(-4.87,102.21) | 0.14(-0.02,0.39) |  | 5.30(-26.43,48.63) | -0.01(-0.09,0.07) |
| Fiji | 0.11(-0.01,0.29) | 0.06(-0.01,0.16) |  | 0.25(-0.03,0.63) | 0.07(-0.01,0.17) |  | 7.46(-20.26,39.93) | -0.06(-0.32,0.20) |
| Finland | 3.45(-0.49,8.81) | 0.05(-0.01,0.13) |  | 6.50(-0.96,16.73) | 0.04(-0.01,0.10) |  | -23.45(-35.19,-9.98) | -1.10(-1.51,-0.70) |
| France | 96.11(-13.73,241.32) | 0.11(-0.02,0.27) |  | 202.70(-30.03,521.94) | 0.09(-0.01,0.24) |  | -14.01(-24.57,-2.60) | -0.48(-0.62,-0.33) |
| Gabon | 0.50(-0.08,1.32) | 0.12(-0.02,0.32) |  | 0.82(-0.13,2.17) | 0.14(-0.02,0.37) |  | 14.45(-16.24,58.29) | 0.28(0.11,0.44) |
| Gambia | 0.22(-0.03,0.58) | 0.13(-0.02,0.34) |  | 1.13(-0.17,2.94) | 0.20(-0.03,0.52) |  | 50.77(2.29,127.67) | 1.26(1.18,1.35) |
| Georgia | 2.56(-0.38,6.69) | 0.05(-0.01,0.13) |  | 8.45(-1.26,20.89) | 0.13(-0.02,0.31) |  | 157.67(93.44,275.57) | 3.22(2.39,4.05) |
| Germany | 68.04(-9.66,172.52) | 0.05(-0.01,0.13) |  | 222.47(-32.44,578.15) | 0.08(-0.01,0.22) |  | 60.60(34.90,88.69) | 2.08(1.76,2.41) |
| Ghana | 2.75(-0.44,6.84) | 0.09(-0.01,0.23) |  | 8.08(-1.17,20.66) | 0.10(-0.01,0.27) |  | 10.07(-22.70,57.16) | -0.06(-0.28,0.16) |
| Greece | 12.11(-1.78,31.01) | 0.09(-0.01,0.24) |  | 39.56(-5.80,99.69) | 0.11(-0.02,0.27) |  | 18.16(4.71,32.66) | 0.48(0.29,0.68) |
| Greenland | 0.02(-0.00,0.05) | 0.11(-0.01,0.27) |  | 0.04(-0.01,0.10) | 0.09(-0.01,0.24) |  | -12.83(-30.98,12.85) | -0.16(-0.27,-0.04) |
| Grenada | 0.17(-0.03,0.43) | 0.19(-0.03,0.49) |  | 0.19(-0.03,0.49) | 0.25(-0.04,0.64) |  | 34.41(3.44,69.99) | 0.75(0.26,1.24) |
| Guam | 0.02(-0.00,0.04) | 0.05(-0.01,0.14) |  | 0.03(-0.00,0.09) | 0.01(-0.00,0.03) |  | -74.02(-79.08,-69.17) | -3.37(-3.88,-2.86) |
| Guatemala | 5.26(-0.77,13.44) | 0.34(-0.05,0.86) |  | 23.92(-3.53,57.41) | 0.29(-0.04,0.71) |  | -14.04(-31.23,6.60) | -0.51(-0.73,-0.29) |
| Guinea | 2.84(-0.45,8.10) | 0.13(-0.02,0.38) |  | 7.12(-1.08,17.78) | 0.20(-0.03,0.51) |  | 51.63(11.81,129.84) | 1.36(1.20,1.51) |
| Guinea-Bissau | 0.27(-0.04,0.73) | 0.14(-0.02,0.39) |  | 0.56(-0.09,1.50) | 0.20(-0.03,0.53) |  | 41.39(2.04,109.60) | 1.15(1.03,1.27) |
| Guyana | 0.43(-0.06,1.08) | 0.16(-0.02,0.41) |  | 0.93(-0.14,2.35) | 0.22(-0.03,0.56) |  | 36.41(9.67,71.09) | 0.98(0.49,1.47) |
| Haiti | 5.57(-0.84,14.66) | 0.33(-0.05,0.86) |  | 13.65(-2.08,35.88) | 0.37(-0.06,0.95) |  | 11.09(-20.14,55.24) | 0.38(0.25,0.51) |
| Honduras | 3.26(-0.55,8.13) | 0.25(-0.04,0.63) |  | 18.38(-2.73,46.99) | 0.47(-0.07,1.20) |  | 88.10(18.54,172.96) | 2.03(1.61,2.47) |
| Hungary | 5.65(-0.83,14.47) | 0.05(-0.01,0.12) |  | 11.36(-1.77,29.56) | 0.05(-0.01,0.13) |  | 9.87(-10.74,38.00) | 0.54(0.28,0.80) |
| Iceland | 0.30(-0.04,0.75) | 0.09(-0.01,0.24) |  | 0.88(-0.12,2.25) | 0.12(-0.02,0.30) |  | 24.15(6.81,44.87) | 1.08(0.81,1.35) |
| India | 237.39(-35.04,632.21) | 0.09(-0.01,0.24) |  | 1352.04(-217.38,3419.69) | 0.17(-0.03,0.43) |  | 83.64(31.58,154.68) | 2.36(2.04,2.68) |
| Indonesia | 46.73(-7.34,128.80) | 0.08(-0.01,0.23) |  | 173.36(-29.06,428.14) | 0.16(-0.03,0.40) |  | 88.31(37.18,165.66) | 2.04(1.83,2.25) |
| Iran (Islamic Republic of) | 22.09(-3.44,55.37) | 0.18(-0.03,0.44) |  | 111.92(-16.74,273.91) | 0.19(-0.03,0.47) |  | 8.77(-16.33,43.70) | 0.23(0.13,0.33) |
| Iraq | 7.11(-1.02,18.47) | 0.10(-0.01,0.27) |  | 21.06(-3.00,54.64) | 0.16(-0.02,0.42) |  | 60.39(9.63,161.71) | 1.05(0.87,1.24) |
| Ireland | 3.86(-0.57,9.77) | 0.11(-0.02,0.27) |  | 8.79(-1.39,22.48) | 0.10(-0.02,0.26) |  | -6.27(-21.73,10.48) | 0.09(-0.20,0.38) |
| Israel | 3.36(-0.48,8.80) | 0.08(-0.01,0.22) |  | 10.60(-1.58,27.95) | 0.07(-0.01,0.19) |  | -14.36(-27.50,-1.41) | -0.41(-0.59,-0.22) |
| Italy | 60.55(-9.49,155.02) | 0.08(-0.01,0.19) |  | 218.30(-33.70,556.70) | 0.10(-0.02,0.25) |  | 31.53(14.13,49.23) | 1.42(1.22,1.62) |
| Jamaica | 3.33(-0.50,8.72) | 0.18(-0.03,0.47) |  | 7.67(-1.09,19.65) | 0.20(-0.03,0.52) |  | 11.00(-13.38,44.93) | 0.32(0.12,0.52) |
| Japan | 55.75(-8.09,139.08) | 0.04(-0.01,0.10) |  | 189.61(-28.31,483.74) | 0.03(-0.00,0.08) |  | -20.10(-28.73,-14.12) | -1.37(-1.74,-1.00) |
| Jordan | 0.66(-0.10,1.75) | 0.09(-0.01,0.23) |  | 3.10(-0.46,7.73) | 0.09(-0.01,0.21) |  | -2.66(-29.59,27.83) | -0.17(-0.46,0.12) |
| Kazakhstan | 2.36(-0.34,6.36) | 0.02(-0.00,0.07) |  | 4.14(-0.60,10.82) | 0.04(-0.01,0.09) |  | 46.96(1.87,106.80) | 0.75(0.47,1.03) |
| Kenya | 4.27(-0.62,11.46) | 0.09(-0.01,0.23) |  | 11.49(-1.85,30.67) | 0.10(-0.02,0.27) |  | 15.17(-16.98,59.79) | 0.47(0.43,0.51) |
| Kiribati | 0.01(-0.00,0.02) | 0.04(-0.01,0.12) |  | 0.02(-0.00,0.04) | 0.05(-0.01,0.13) |  | 18.45(-11.60,58.05) | 0.45(0.29,0.60) |
| Kuwait | 0.21(-0.03,0.52) | 0.06(-0.01,0.16) |  | 1.53(-0.23,3.85) | 0.08(-0.01,0.19) |  | 25.08(1.93,54.15) | 1.12(0.48,1.77) |
| Kyrgyzstan | 0.76(-0.12,1.97) | 0.03(-0.01,0.09) |  | 1.55(-0.23,3.97) | 0.05(-0.01,0.12) |  | 42.28(9.69,78.01) | 1.34(1.09,1.59) |
| Lao People's Democratic Republic | 1.20(-0.19,3.43) | 0.12(-0.02,0.35) |  | 4.59(-0.67,12.26) | 0.18(-0.03,0.49) |  | 53.38(10.45,119.80) | 1.32(1.20,1.43) |
| Latvia | 1.04(-0.15,2.72) | 0.03(-0.00,0.08) |  | 2.48(-0.37,6.40) | 0.05(-0.01,0.13) |  | 54.11(27.97,86.38) | 1.62(1.43,1.81) |
| Lebanon | 1.59(-0.24,4.42) | 0.11(-0.02,0.30) |  | 5.96(-0.79,15.36) | 0.08(-0.01,0.22) |  | -22.19(-53.21,50.11) | -1.08(-1.24,-0.91) |
| Lesotho | 0.46(-0.07,1.21) | 0.08(-0.01,0.21) |  | 0.77(-0.11,2.11) | 0.12(-0.02,0.34) |  | 55.68(-5.70,117.87) | 2.51(2.06,2.96) |
| Liberia | 0.81(-0.13,2.25) | 0.13(-0.02,0.35) |  | 1.96(-0.30,5.11) | 0.18(-0.03,0.46) |  | 40.13(5.12,100.95) | 1.10(0.99,1.21) |
| Libya | 1.32(-0.19,3.26) | 0.09(-0.01,0.21) |  | 3.63(-0.58,10.00) | 0.10(-0.02,0.28) |  | 19.10(-25.41,85.96) | 1.27(0.98,1.57) |
| Lithuania | 1.37(-0.20,3.61) | 0.03(-0.00,0.09) |  | 3.68(-0.57,9.09) | 0.05(-0.01,0.12) |  | 54.85(29.39,87.12) | 1.46(1.30,1.62) |
| Luxembourg | 0.48(-0.07,1.21) | 0.10(-0.01,0.24) |  | 1.30(-0.19,3.18) | 0.10(-0.01,0.24) |  | 3.25(-14.99,22.02) | 0.45(0.21,0.70) |
| Madagascar | 5.44(-0.74,14.80) | 0.18(-0.02,0.48) |  | 8.67(-1.25,22.76) | 0.18(-0.03,0.48) |  | 0.45(-27.42,41.18) | 0.02(-0.02,0.07) |
| Malawi | 1.86(-0.26,5.06) | 0.10(-0.01,0.27) |  | 4.61(-0.66,11.76) | 0.12(-0.02,0.30) |  | 16.56(-13.44,80.65) | 0.47(0.37,0.57) |
| Malaysia | 4.14(-0.64,10.42) | 0.06(-0.01,0.14) |  | 19.20(-2.84,48.91) | 0.10(-0.02,0.26) |  | 84.61(42.89,157.88) | 1.93(1.40,2.47) |
| Maldives | 0.03(-0.01,0.09) | 0.10(-0.02,0.29) |  | 0.26(-0.03,0.67) | 0.11(-0.01,0.29) |  | 5.97(-31.43,73.57) | -0.06(-0.27,0.15) |
| Mali | 2.10(-0.36,5.63) | 0.11(-0.02,0.30) |  | 6.31(-1.05,16.91) | 0.14(-0.02,0.36) |  | 27.45(-8.32,81.21) | 0.93(0.83,1.03) |
| Malta | 0.67(-0.10,1.66) | 0.19(-0.03,0.46) |  | 2.21(-0.34,5.58) | 0.18(-0.03,0.46) |  | -0.14(-14.76,16.13) | 0.28(0.03,0.53) |
| Marshall Islands | 0.01(-0.00,0.02) | 0.10(-0.01,0.26) |  | 0.01(-0.00,0.04) | 0.10(-0.01,0.27) |  | 4.88(-19.73,33.76) | 0.09(-0.01,0.20) |
| Mauritania | 0.62(-0.09,1.63) | 0.11(-0.01,0.28) |  | 1.90(-0.28,4.86) | 0.14(-0.02,0.36) |  | 33.95(-6.91,93.50) | 0.78(0.71,0.85) |
| Mauritius | 0.31(-0.04,0.78) | 0.07(-0.01,0.18) |  | 1.18(-0.17,3.01) | 0.08(-0.01,0.19) |  | 5.68(-7.69,18.63) | 0.12(-0.08,0.32) |
| Mexico | 58.26(-8.46,147.80) | 0.22(-0.03,0.55) |  | 205.86(-32.61,515.67) | 0.20(-0.03,0.49) |  | -10.04(-19.57,0.86) | -0.35(-0.53,-0.17) |
| Micronesia (Federated States of) | 0.02(-0.00,0.07) | 0.08(-0.01,0.22) |  | 0.04(-0.01,0.09) | 0.10(-0.01,0.25) |  | 14.73(-12.53,48.53) | 0.38(0.32,0.45) |
| Monaco | 0.05(-0.01,0.12) | 0.05(-0.01,0.13) |  | 0.08(-0.01,0.22) | 0.06(-0.01,0.16) |  | 17.59(-14.61,72.00) | 0.57(0.21,0.93) |
| Mongolia | 0.38(-0.05,0.99) | 0.05(-0.01,0.13) |  | 0.64(-0.10,1.68) | 0.05(-0.01,0.13) |  | -1.57(-29.30,32.39) | -0.52(-0.76,-0.29) |
| Montenegro | 0.70(-0.10,1.78) | 0.13(-0.02,0.33) |  | 1.83(-0.28,4.66) | 0.24(-0.04,0.61) |  | 88.26(31.62,158.98) | 2.37(1.94,2.80) |
| Morocco | 8.73(-1.33,23.65) | 0.09(-0.01,0.24) |  | 28.99(-4.61,75.67) | 0.13(-0.02,0.33) |  | 46.08(2.31,118.55) | 1.29(1.21,1.38) |
| Mozambique | 5.01(-0.75,14.01) | 0.17(-0.02,0.45) |  | 11.40(-1.97,29.25) | 0.20(-0.04,0.52) |  | 23.74(-15.12,83.23) | 1.01(0.90,1.13) |
| Myanmar | 13.52(-2.05,36.53) | 0.11(-0.02,0.29) |  | 51.69(-7.21,128.13) | 0.16(-0.02,0.40) |  | 49.55(3.80,114.24) | 1.07(0.93,1.21) |
| Namibia | 0.26(-0.04,0.66) | 0.08(-0.01,0.20) |  | 0.79(-0.13,2.00) | 0.10(-0.02,0.26) |  | 32.60(-1.66,77.83) | 1.01(0.75,1.28) |
| Nauru | 0.00(-0.00,0.00) | 0.07(-0.01,0.18) |  | 0.00(-0.00,0.01) | 0.09(-0.01,0.27) |  | 27.89(-15.78,138.99) | 0.86(0.79,0.93) |
| Nepal | 6.85(-1.02,19.44) | 0.14(-0.02,0.38) |  | 38.47(-6.43,102.04) | 0.27(-0.04,0.71) |  | 98.54(40.41,201.80) | 2.38(2.15,2.62) |
| Netherlands | 21.83(-3.11,55.69) | 0.11(-0.02,0.28) |  | 41.16(-6.10,105.60) | 0.10(-0.01,0.25) |  | -9.80(-21.94,1.41) | -0.47(-0.54,-0.40) |
| New Zealand | 5.60(-0.84,13.89) | 0.16(-0.02,0.39) |  | 17.40(-2.64,44.00) | 0.18(-0.03,0.45) |  | 12.67(-0.05,25.31) | 0.59(0.49,0.68) |
| Nicaragua | 2.23(-0.33,5.45) | 0.20(-0.03,0.49) |  | 6.89(-1.09,16.93) | 0.19(-0.03,0.47) |  | -4.06(-27.02,23.31) | 0.19(-0.13,0.51) |
| Niger | 1.54(-0.23,4.17) | 0.13(-0.02,0.36) |  | 6.70(-0.96,17.77) | 0.18(-0.03,0.47) |  | 42.80(1.23,97.47) | 1.15(1.09,1.22) |
| Nigeria | 27.00(-4.36,73.37) | 0.11(-0.02,0.29) |  | 51.05(-8.80,128.79) | 0.11(-0.02,0.27) |  | -1.25(-27.22,43.61) | -0.48(-0.68,-0.29) |
| Niue | 0.00(-0.00,0.00) | 0.06(-0.01,0.17) |  | 0.00(-0.00,0.00) | 0.06(-0.01,0.15) |  | -9.74(-30.78,12.58) | -0.40(-0.47,-0.33) |
| North Macedonia | 0.95(-0.14,2.48) | 0.06(-0.01,0.17) |  | 2.60(-0.40,6.65) | 0.13(-0.02,0.34) |  | 103.14(43.18,170.16) | 2.10(1.37,2.83) |
| Northern Mariana Islands | 0.00(-0.00,0.01) | 0.04(-0.01,0.11) |  | 0.01(-0.00,0.03) | 0.05(-0.01,0.13) |  | 12.41(-12.16,40.24) | 0.27(-0.05,0.60) |
| Norway | 6.60(-0.97,16.53) | 0.08(-0.01,0.21) |  | 11.89(-1.83,30.11) | 0.09(-0.01,0.23) |  | 6.23(-8.01,20.30) | 0.12(-0.25,0.50) |
| Oman | 0.53(-0.08,1.39) | 0.13(-0.02,0.34) |  | 1.29(-0.19,3.29) | 0.14(-0.02,0.38) |  | 12.26(-27.25,78.03) | 0.83(0.43,1.23) |
| Pakistan | 45.30(-7.21,124.11) | 0.12(-0.02,0.34) |  | 150.96(-23.32,380.50) | 0.23(-0.04,0.56) |  | 86.67(36.64,167.26) | 1.87(1.50,2.23) |
| Palau | 0.00(-0.00,0.00) | 0.03(-0.00,0.09) |  | 0.00(-0.00,0.01) | 0.03(-0.00,0.08) |  | -5.43(-27.49,18.93) | -0.00(-0.10,0.09) |
| Palestine | 1.33(-0.21,3.49) | 0.24(-0.04,0.63) |  | 3.06(-0.48,7.92) | 0.25(-0.04,0.66) |  | 5.86(-20.45,47.52) | 0.02(-0.28,0.31) |
| Panama | 1.76(-0.27,4.51) | 0.14(-0.02,0.36) |  | 7.87(-1.11,19.90) | 0.16(-0.02,0.41) |  | 14.77(-9.19,41.35) | 0.49(0.30,0.68) |
| Papua New Guinea | 0.42(-0.06,1.15) | 0.05(-0.01,0.15) |  | 1.59(-0.25,4.31) | 0.07(-0.01,0.18) |  | 30.33(-8.29,84.08) | 0.86(0.79,0.93) |
| Paraguay | 2.20(-0.34,5.73) | 0.12(-0.02,0.31) |  | 8.53(-1.16,21.46) | 0.17(-0.02,0.42) |  | 40.64(3.44,92.52) | 1.42(1.24,1.59) |
| Peru | 10.28(-1.53,27.63) | 0.10(-0.02,0.27) |  | 33.91(-4.72,89.81) | 0.10(-0.01,0.27) |  | -1.03(-25.77,38.14) | -0.36(-0.65,-0.07) |
| Philippines | 9.26(-1.30,23.43) | 0.07(-0.01,0.19) |  | 42.72(-6.02,109.34) | 0.08(-0.01,0.22) |  | 17.76(-4.99,40.27) | 1.06(0.86,1.27) |
| Poland | 40.05(-5.61,100.57) | 0.11(-0.02,0.27) |  | 77.16(-11.07,196.37) | 0.09(-0.01,0.24) |  | -12.30(-23.39,0.58) | -0.05(-0.65,0.54) |
| Portugal | 15.93(-2.42,40.33) | 0.15(-0.02,0.37) |  | 40.88(-6.28,101.94) | 0.12(-0.02,0.30) |  | -18.14(-27.81,-5.86) | -0.83(-1.08,-0.58) |
| Puerto Rico | 2.90(-0.40,7.41) | 0.10(-0.01,0.26) |  | 7.04(-0.95,17.56) | 0.07(-0.01,0.17) |  | -33.76(-46.11,-20.77) | -1.57(-1.79,-1.34) |
| Qatar | 0.04(-0.01,0.12) | 0.13(-0.02,0.37) |  | 0.18(-0.02,0.50) | 0.08(-0.01,0.23) |  | -40.31(-60.58,-18.55) | -2.54(-3.35,-1.72) |
| Republic of Korea | 13.72(-1.81,38.65) | 0.08(-0.01,0.23) |  | 78.67(-12.00,196.46) | 0.09(-0.01,0.22) |  | 6.33(-49.29,51.26) | 0.79(0.50,1.09) |
| Republic of Moldova | 1.24(-0.18,3.16) | 0.04(-0.01,0.11) |  | 3.25(-0.54,8.16) | 0.05(-0.01,0.13) |  | 18.75(-0.52,42.06) | 0.31(0.15,0.47) |
| Romania | 10.11(-1.47,27.16) | 0.05(-0.01,0.14) |  | 24.55(-3.76,61.18) | 0.06(-0.01,0.14) |  | 5.83(-13.84,30.63) | -0.07(-0.22,0.08) |
| Russian Federation | 46.36(-6.82,121.01) | 0.04(-0.01,0.09) |  | 128.50(-18.62,314.82) | 0.05(-0.01,0.13) |  | 49.40(23.48,75.25) | 1.32(1.10,1.54) |
| Rwanda | 2.22(-0.32,5.84) | 0.15(-0.02,0.40) |  | 3.79(-0.49,10.11) | 0.12(-0.02,0.32) |  | -21.38(-47.18,13.04) | -1.38(-1.62,-1.13) |
| Saint Kitts and Nevis | 0.06(-0.01,0.16) | 0.21(-0.03,0.53) |  | 0.08(-0.01,0.20) | 0.20(-0.03,0.50) |  | -3.43(-20.41,13.57) | 0.12(-0.16,0.41) |
| Saint Lucia | 0.24(-0.03,0.59) | 0.41(-0.06,1.04) |  | 0.62(-0.09,1.65) | 0.28(-0.04,0.76) |  | -30.73(-43.94,-16.96) | -2.06(-2.39,-1.74) |
| Saint Vincent and the Grenadines | 0.20(-0.03,0.51) | 0.36(-0.05,0.89) |  | 0.38(-0.05,0.94) | 0.35(-0.05,0.85) |  | -2.20(-14.75,12.73) | -0.08(-0.28,0.11) |
| Samoa | 0.04(-0.01,0.11) | 0.08(-0.01,0.20) |  | 0.07(-0.01,0.18) | 0.07(-0.01,0.19) |  | -5.52(-27.66,20.55) | -0.26(-0.30,-0.21) |
| San Marino | 0.03(-0.00,0.07) | 0.07(-0.01,0.19) |  | 0.05(-0.01,0.13) | 0.04(-0.01,0.10) |  | -45.02(-62.89,-16.20) | -1.06(-1.49,-0.63) |
| Sao Tome and Principe | 0.04(-0.01,0.11) | 0.09(-0.01,0.24) |  | 0.09(-0.02,0.23) | 0.15(-0.03,0.38) |  | 72.10(5.20,157.60) | 2.09(2.00,2.18) |
| Saudi Arabia | 3.52(-0.52,9.38) | 0.10(-0.01,0.27) |  | 7.82(-1.16,19.67) | 0.11(-0.02,0.29) |  | 13.19(-18.64,61.68) | 0.30(0.13,0.48) |
| Senegal | 2.23(-0.39,5.96) | 0.12(-0.02,0.32) |  | 7.19(-1.19,18.97) | 0.16(-0.03,0.42) |  | 32.10(-10.36,98.23) | 0.75(0.67,0.83) |
| Serbia | 5.48(-0.82,14.44) | 0.08(-0.01,0.21) |  | 13.41(-1.99,34.33) | 0.08(-0.01,0.20) |  | -3.80(-24.58,31.61) | -0.55(-0.87,-0.24) |
| Seychelles | 0.03(-0.00,0.08) | 0.06(-0.01,0.15) |  | 0.06(-0.01,0.16) | 0.07(-0.01,0.20) |  | 22.79(-2.01,53.75) | 0.87(0.64,1.10) |
| Sierra Leone | 1.19(-0.20,3.04) | 0.09(-0.02,0.23) |  | 2.86(-0.48,7.31) | 0.14(-0.02,0.35) |  | 49.44(6.08,124.90) | 1.36(1.12,1.61) |
| Singapore | 0.93(-0.13,2.29) | 0.06(-0.01,0.14) |  | 2.98(-0.44,7.69) | 0.04(-0.01,0.10) |  | -34.44(-42.83,-27.42) | -1.73(-2.02,-1.44) |
| Slovakia | 3.44(-0.46,9.21) | 0.06(-0.01,0.17) |  | 7.63(-1.05,19.52) | 0.08(-0.01,0.21) |  | 25.67(-7.66,61.83) | 1.01(0.81,1.21) |
| Slovenia | 0.85(-0.12,2.22) | 0.04(-0.01,0.10) |  | 2.70(-0.39,6.86) | 0.05(-0.01,0.12) |  | 27.43(6.13,54.53) | 1.08(0.55,1.61) |
| Solomon Islands | 0.07(-0.01,0.19) | 0.12(-0.02,0.35) |  | 0.22(-0.03,0.62) | 0.14(-0.02,0.39) |  | 12.74(-14.86,48.77) | 0.33(0.23,0.43) |
| Somalia | 1.76(-0.28,4.84) | 0.16(-0.03,0.46) |  | 3.53(-0.62,9.46) | 0.14(-0.02,0.36) |  | -15.36(-37.32,15.80) | -0.38(-0.45,-0.31) |
| South Africa | 7.28(-1.05,19.64) | 0.05(-0.01,0.13) |  | 24.44(-3.52,59.88) | 0.08(-0.01,0.20) |  | 68.45(24.39,123.80) | 1.76(1.26,2.25) |
| South Sudan | 2.20(-0.30,6.53) | 0.14(-0.02,0.39) |  | 2.52(-0.34,6.82) | 0.13(-0.02,0.34) |  | -6.12(-30.04,24.09) | -0.25(-0.31,-0.19) |
| Spain | 60.46(-8.89,152.20) | 0.12(-0.02,0.30) |  | 191.98(-28.34,490.91) | 0.13(-0.02,0.32) |  | 5.57(-8.22,18.58) | 0.24(0.07,0.41) |
| Sri Lanka | 2.41(-0.34,6.38) | 0.04(-0.01,0.11) |  | 10.87(-1.38,27.50) | 0.06(-0.01,0.14) |  | 34.96(-10.16,87.43) | 1.63(1.30,1.96) |
| Sudan | 7.37(-1.25,19.61) | 0.14(-0.02,0.37) |  | 19.24(-3.03,50.70) | 0.16(-0.03,0.42) |  | 19.16(-17.25,95.04) | 0.50(0.48,0.53) |
| Suriname | 0.40(-0.06,1.02) | 0.18(-0.03,0.47) |  | 1.04(-0.19,2.65) | 0.19(-0.03,0.49) |  | 4.18(-22.63,39.83) | 0.36(0.15,0.56) |
| Sweden | 7.24(-1.05,18.88) | 0.04(-0.01,0.11) |  | 29.91(-4.28,76.60) | 0.10(-0.01,0.25) |  | 134.76(102.36,172.07) | 3.24(2.88,3.61) |
| Switzerland | 5.39(-0.76,13.78) | 0.05(-0.01,0.12) |  | 15.39(-2.25,39.95) | 0.06(-0.01,0.15) |  | 26.88(8.91,45.85) | 1.05(0.91,1.18) |
| Syrian Arab Republic | 6.28(-0.89,16.31) | 0.17(-0.02,0.45) |  | 13.38(-1.99,35.06) | 0.20(-0.03,0.54) |  | 16.96(-23.29,79.52) | 0.16(-0.11,0.43) |
| Taiwan (Province of China) | 10.13(-1.45,25.84) | 0.13(-0.02,0.32) |  | 50.37(-7.38,128.39) | 0.11(-0.02,0.27) |  | -17.36(-27.18,-6.60) | -0.89(-1.33,-0.45) |
| Tajikistan | 0.63(-0.10,1.72) | 0.03(-0.00,0.08) |  | 1.09(-0.17,2.74) | 0.03(-0.01,0.08) |  | 14.24(-26.57,66.11) | 0.18(-0.25,0.61) |
| Thailand | 14.92(-2.30,42.71) | 0.07(-0.01,0.20) |  | 78.47(-11.60,196.88) | 0.07(-0.01,0.18) |  | 0.16(-32.45,39.52) | -0.60(-0.87,-0.34) |
| Timor-Leste | 0.16(-0.02,0.44) | 0.12(-0.02,0.32) |  | 0.85(-0.13,2.14) | 0.18(-0.03,0.45) |  | 53.49(6.12,143.06) | 1.48(1.41,1.55) |
| Togo | 0.65(-0.11,1.64) | 0.10(-0.02,0.27) |  | 2.39(-0.36,5.86) | 0.15(-0.02,0.37) |  | 42.75(1.59,105.59) | 1.09(0.97,1.21) |
| Tokelau | 0.00(-0.00,0.00) | 0.08(-0.01,0.21) |  | 0.00(-0.00,0.00) | 0.07(-0.01,0.18) |  | -12.27(-39.07,23.86) | -0.39(-0.45,-0.33) |
| Tonga | 0.02(-0.00,0.06) | 0.06(-0.01,0.17) |  | 0.04(-0.01,0.11) | 0.06(-0.01,0.16) |  | -5.64(-32.81,28.88) | -0.18(-0.34,-0.02) |
| Trinidad and Tobago | 0.58(-0.08,1.48) | 0.12(-0.02,0.31) |  | 1.54(-0.23,4.00) | 0.09(-0.01,0.23) |  | -25.47(-43.30,-8.71) | -0.81(-0.91,-0.71) |
| Tunisia | 3.32(-0.47,8.88) | 0.13(-0.02,0.33) |  | 17.04(-2.44,43.00) | 0.17(-0.02,0.43) |  | 35.84(-13.48,98.75) | 0.80(0.62,0.98) |
| Turkey | 0.41(-0.06,1.09) | 0.03(-0.00,0.08) |  | 1.39(-0.23,3.46) | 0.05(-0.01,0.12) |  | 48.94(12.20,95.75) | 0.91(0.34,1.48) |
| Turkmenistan | 0.00(-0.00,0.01) | 0.11(-0.02,0.29) |  | 0.01(-0.00,0.02) | 0.10(-0.01,0.26) |  | -6.50(-27.36,17.96) | 0.87(0.60,1.14) |
| Tuvalu | 20.44(-2.99,54.46) | 0.09(-0.01,0.23) |  | 73.41(-10.57,192.10) | 0.10(-0.01,0.26) |  | 13.20(-20.77,56.82) | -0.27(-0.39,-0.15) |
| Uganda | 4.03(-0.65,11.27) | 0.11(-0.02,0.31) |  | 9.47(-1.40,25.68) | 0.11(-0.02,0.30) |  | 3.85(-29.36,63.71) | 0.11(0.02,0.19) |
| Ukraine | 19.08(-2.59,48.66) | 0.03(-0.00,0.09) |  | 34.64(-4.89,93.22) | 0.04(-0.01,0.12) |  | 28.96(-1.56,68.01) | 0.41(0.08,0.74) |
| United Arab Emirates | 0.15(-0.02,0.41) | 0.08(-0.01,0.21) |  | 0.53(-0.07,1.38) | 0.08(-0.01,0.22) |  | 0.81(-38.86,49.56) | 2.79(1.61,3.99) |
| United Kingdom | 62.51(-9.28,155.56) | 0.07(-0.01,0.17) |  | 137.92(-20.34,346.53) | 0.08(-0.01,0.21) |  | 22.94(11.98,29.27) | 0.97(0.82,1.12) |
| United Republic of Tanzania | 6.26(-0.80,17.25) | 0.11(-0.01,0.30) |  | 14.07(-2.37,37.94) | 0.09(-0.02,0.25) |  | -17.71(-40.40,23.16) | -0.95(-1.08,-0.83) |
| United States of America | 215.51(-32.49,528.17) | 0.06(-0.01,0.16) |  | 543.49(-82.15,1387.91) | 0.08(-0.01,0.21) |  | 25.32(12.83,37.32) | 0.82(0.70,0.94) |
| United States Virgin Islands | 0.06(-0.01,0.15) | 0.12(-0.02,0.32) |  | 0.13(-0.02,0.33) | 0.09(-0.01,0.23) |  | -28.06(-46.43,-1.94) | -0.94(-1.30,-0.58) |
| Uruguay | 1.79(-0.25,4.55) | 0.05(-0.01,0.12) |  | 6.44(-0.96,16.11) | 0.09(-0.01,0.22) |  | 82.10(59.45,105.33) | 2.15(1.94,2.36) |
| Uzbekistan | 1.50(-0.22,4.61) | 0.02(-0.00,0.05) |  | 4.29(-0.61,10.94) | 0.03(-0.00,0.07) |  | 59.39(-13.84,184.16) | 1.80(1.57,2.04) |
| Vanuatu | 0.03(-0.00,0.08) | 0.10(-0.02,0.27) |  | 0.08(-0.01,0.23) | 0.10(-0.02,0.28) |  | 1.11(-19.01,34.25) | 0.04(-0.09,0.16) |
| Venezuela (Bolivarian Republic of) | 10.33(-1.46,25.99) | 0.15(-0.02,0.37) |  | 45.48(-6.49,112.80) | 0.17(-0.02,0.43) |  | 18.08(-11.96,51.65) | 0.36(0.18,0.54) |
| Viet Nam | 23.92(-3.65,61.61) | 0.08(-0.01,0.21) |  | 90.00(-13.47,221.78) | 0.13(-0.02,0.32) |  | 62.38(17.56,122.41) | 1.38(1.19,1.57) |
| Yemen | 4.42(-0.71,11.96) | 0.18(-0.03,0.47) |  | 18.45(-3.05,49.17) | 0.24(-0.04,0.64) |  | 37.46(-10.77,110.97) | 0.99(0.96,1.03) |
| Zambia | 2.34(-0.35,6.13) | 0.16(-0.02,0.42) |  | 6.88(-0.95,18.53) | 0.19(-0.03,0.52) |  | 23.32(-19.74,83.27) | 0.91(0.71,1.12) |
| Zimbabwe | 1.91(-0.28,4.65) | 0.09(-0.01,0.22) |  | 3.64(-0.56,9.47) | 0.11(-0.02,0.28) |  | 19.00(-23.65,63.46) | 1.10(0.80,1.39) |
