# Supplementary material for: The burden and trend prediction of atrial fibrillation and flutter associated with lead exposure: insights from the global burden of disease study 2021
Source: Front Cardiovasc Med. 2025 Aug 12;12:1638747. doi: 10.3389/fcvm.2025.1638747 (PMC12378705; doi:10.3389/fcvm.2025.1638747)
Supplement: Supplementary file 3 [file Table2.docx]

Table S2: DALYs of lead exposure-related atrial fibrillation and flutter between 1990 and 2021 at the national level

| location | 1990 | |  | 2021 | |  | 1990-2021 | |
| --- | --- | --- | --- | --- | --- | --- | --- | --- |
|  | DALYs cases | DALYs rate |  | DALYs cases | DALYs rate |  | Rate change | EAPC |
| Afghanistan | 194.20(-31.39,542.59) | 3.60(-0.59,10.02) |  | 309.73(-46.48,813.67) | 4.61(-0.70,12.06) |  | 27.97(4.78,58.51) | -2.06(-2.22,-1.90) |
| Albania | 33.48(-4.30,88.42) | 1.88(-0.25,4.94) |  | 98.10(-13.61,253.34) | 2.30(-0.32,5.90) |  | 22.24(1.80,50.97) | 4.66(4.49,4.84) |
| Algeria | 229.73(-30.34,593.73) | 3.09(-0.42,7.61) |  | 810.53(-100.42,2003.62) | 3.42(-0.44,8.46) |  | 10.65(-11.88,43.60) | 2.51(2.42,2.59) |
| American Samoa | 0.13(-0.02,0.34) | 0.88(-0.11,2.22) |  | 0.36(-0.05,0.94) | 0.94(-0.12,2.45) |  | 7.51(-6.96,25.06) | 3.28(3.22,3.34) |
| Andorra | 0.73(-0.09,1.88) | 1.42(-0.19,3.60) |  | 2.23(-0.29,5.87) | 1.34(-0.17,3.59) |  | -5.62(-25.44,14.74) | 2.12(1.75,2.48) |
| Angola | 69.55(-9.97,180.03) | 2.48(-0.37,6.34) |  | 235.19(-36.22,600.53) | 3.03(-0.47,7.71) |  | 22.42(-4.64,57.26) | 0.13(0.07,0.19) |
| Antigua and Barbuda | 1.87(-0.25,4.80) | 3.26(-0.44,8.36) |  | 2.81(-0.38,7.23) | 3.14(-0.43,8.17) |  | -3.64(-13.64,8.23) | -0.05(-0.29,0.19) |
| Argentina | 326.18(-40.62,840.31) | 1.09(-0.14,2.81) |  | 679.89(-90.48,1743.92) | 1.17(-0.16,2.99) |  | 6.80(-6.68,21.45) | 1.74(1.47,2.02) |
| Armenia | 24.27(-2.93,65.18) | 1.01(-0.12,2.68) |  | 63.79(-7.90,169.83) | 1.46(-0.18,3.86) |  | 44.28(26.87,65.82) | 4.05(3.80,4.30) |
| Australia | 758.03(-101.83,1970.44) | 4.05(-0.55,10.44) |  | 1910.49(-250.14,4901.56) | 3.72(-0.48,9.58) |  | -8.16(-20.63,5.01) | 1.77(1.45,2.09) |
| Austria | 209.07(-28.44,531.22) | 1.70(-0.23,4.35) |  | 544.34(-76.12,1380.85) | 2.50(-0.35,6.38) |  | 46.92(28.19,65.29) | 2.89(2.58,3.21) |
| Azerbaijan | 43.59(-5.53,118.25) | 1.00(-0.13,2.69) |  | 98.41(-11.93,266.72) | 1.18(-0.15,3.19) |  | 18.02(1.61,36.13) | 1.57(1.45,1.69) |
| Bahamas | 2.82(-0.38,7.55) | 2.08(-0.28,5.58) |  | 7.18(-0.97,18.46) | 2.10(-0.29,5.37) |  | 1.02(-13.27,17.25) | 1.62(1.44,1.79) |
| Bahrain | 2.40(-0.33,6.35) | 2.58(-0.36,7.02) |  | 9.60(-1.24,25.33) | 2.25(-0.33,5.98) |  | -12.71(-31.76,7.61) | 0.02(-0.33,0.36) |
| Bangladesh | 1587.97(-223.87,3896.50) | 4.20(-0.60,10.48) |  | 5719.29(-810.97,14549.23) | 5.27(-0.76,13.36) |  | 25.54(4.72,60.20) | 3.00(2.76,3.24) |
| Barbados | 7.89(-1.02,19.85) | 2.58(-0.33,6.52) |  | 13.07(-1.66,33.33) | 2.50(-0.32,6.39) |  | -3.12(-16.12,10.68) | 0.72(0.56,0.88) |
| Belarus | 129.33(-17.21,337.00) | 1.03(-0.14,2.70) |  | 232.81(-30.86,601.98) | 1.41(-0.19,3.64) |  | 36.38(16.26,65.30) | 2.28(2.11,2.45) |
| Belgium | 549.51(-73.75,1430.58) | 3.48(-0.47,8.99) |  | 849.26(-118.20,2145.34) | 3.03(-0.41,7.70) |  | -12.79(-21.37,-1.63) | 1.11(0.87,1.35) |
| Belize | 2.59(-0.35,6.66) | 2.86(-0.39,7.34) |  | 7.07(-0.89,18.37) | 2.79(-0.36,7.19) |  | -2.44(-16.92,14.58) | 0.28(0.08,0.49) |
| Benin | 33.76(-4.85,84.55) | 2.05(-0.30,5.09) |  | 89.95(-12.39,230.94) | 2.43(-0.34,6.21) |  | 18.78(-1.96,45.05) | -0.06(-0.10,-0.03) |
| Bermuda | 1.13(-0.15,2.90) | 2.02(-0.26,5.23) |  | 2.30(-0.29,5.97) | 1.48(-0.19,3.87) |  | -26.69(-37.30,-13.25) | 2.07(1.98,2.16) |
| Bhutan | 7.01(-0.98,18.72) | 4.11(-0.59,10.88) |  | 28.54(-3.99,71.71) | 5.42(-0.77,13.54) |  | 31.81(4.75,60.93) | 4.02(3.84,4.21) |
| Bolivia (Plurinational State of) | 99.07(-13.91,265.90) | 3.79(-0.54,10.20) |  | 336.14(-44.62,892.39) | 4.45(-0.60,11.83) |  | 17.46(-1.63,45.57) | 2.27(2.16,2.38) |
| Bosnia and Herzegovina | 80.45(-10.32,202.27) | 2.27(-0.29,5.75) |  | 168.21(-21.77,449.07) | 2.61(-0.33,6.90) |  | 14.81(-4.43,41.85) | 3.39(3.11,3.67) |
| Botswana | 11.53(-1.65,30.06) | 2.78(-0.40,7.25) |  | 26.93(-3.85,70.41) | 2.51(-0.35,6.44) |  | -9.70(-30.30,16.96) | 0.83(0.73,0.94) |
| Brazil | 2699.01(-341.33,6910.61) | 3.73(-0.48,9.43) |  | 8525.90(-1086.29,21600.90) | 3.55(-0.45,8.98) |  | -4.92(-8.89,-0.48) | 2.49(2.38,2.60) |
| Brunei Darussalam | 2.74(-0.38,7.04) | 3.19(-0.45,8.14) |  | 5.83(-0.75,15.37) | 2.39(-0.31,6.30) |  | -25.04(-39.57,-6.44) | 0.82(0.67,0.98) |
| Bulgaria | 193.36(-25.10,530.26) | 1.84(-0.24,5.04) |  | 333.47(-42.14,857.78) | 2.21(-0.28,5.69) |  | 19.72(1.90,42.48) | 2.53(2.39,2.66) |
| Burkina Faso | 104.36(-14.74,273.18) | 3.36(-0.49,8.95) |  | 318.67(-47.73,828.01) | 4.81(-0.75,12.76) |  | 43.37(16.42,78.04) | 0.92(0.83,1.02) |
| Burundi | 56.55(-8.42,150.67) | 3.04(-0.46,8.20) |  | 97.58(-14.00,266.59) | 2.90(-0.42,7.95) |  | -4.56(-25.80,21.97) | -1.34(-1.55,-1.13) |
| Cabo Verde | 4.60(-0.65,11.68) | 1.93(-0.27,4.85) |  | 10.42(-1.47,26.39) | 2.54(-0.36,6.41) |  | 31.58(-7.60,86.50) | 1.18(1.01,1.34) |
| Cambodia | 76.67(-10.08,204.49) | 2.23(-0.31,5.92) |  | 287.06(-35.82,761.29) | 3.14(-0.40,8.33) |  | 41.09(18.98,68.44) | 2.83(2.77,2.90) |
| Cameroon | 92.55(-12.26,240.83) | 2.92(-0.40,7.65) |  | 298.45(-40.61,756.42) | 3.56(-0.49,9.10) |  | 21.95(-0.49,61.68) | 0.02(-0.07,0.11) |
| Canada | 624.19(-81.46,1644.48) | 1.92(-0.25,5.07) |  | 1288.22(-166.96,3400.23) | 1.61(-0.21,4.25) |  | -16.39(-24.91,-7.55) | 1.33(1.24,1.42) |
| Central African Republic | 24.92(-3.78,63.36) | 3.01(-0.49,7.66) |  | 50.99(-7.80,136.45) | 3.58(-0.57,9.64) |  | 18.90(-2.69,43.87) | 0.02(-0.09,0.13) |
| Chad | 66.27(-9.84,171.88) | 2.88(-0.44,7.67) |  | 165.91(-22.63,414.71) | 4.20(-0.58,10.51) |  | 45.99(20.13,86.28) | -0.55(-0.65,-0.44) |
| Chile | 68.71(-8.52,177.33) | 0.75(-0.09,1.94) |  | 222.76(-29.26,556.83) | 0.85(-0.11,2.12) |  | 12.48(-0.66,27.80) | 3.31(2.96,3.66) |
| China | 17147.29(-2181.29,44458.64) | 2.92(-0.38,7.60) |  | 56945.30(-7718.89,146211.01) | 3.09(-0.43,8.01) |  | 5.93(-13.21,28.42) | 3.34(3.26,3.42) |
| Colombia | 570.36(-74.00,1456.58) | 3.93(-0.52,9.93) |  | 1724.15(-217.19,4291.43) | 3.06(-0.39,7.64) |  | -22.29(-31.99,-10.60) | 2.25(2.19,2.32) |
| Comoros | 4.55(-0.66,11.80) | 3.15(-0.47,8.13) |  | 9.17(-1.32,23.74) | 2.49(-0.36,6.48) |  | -21.18(-37.72,-1.37) | 0.70(0.63,0.77) |
| Congo | 19.66(-2.96,52.06) | 2.49(-0.38,6.61) |  | 44.88(-6.55,117.74) | 2.53(-0.38,6.62) |  | 1.64(-20.86,28.39) | -0.12(-0.23,-0.00) |
| Cook Islands | 0.11(-0.01,0.29) | 1.16(-0.15,2.99) |  | 0.23(-0.03,0.61) | 0.90(-0.12,2.39) |  | -22.39(-35.55,-8.14) | 2.62(2.50,2.74) |
| Costa Rica | 58.21(-7.86,148.62) | 3.60(-0.49,9.18) |  | 194.65(-25.73,487.41) | 3.48(-0.46,8.69) |  | -3.22(-14.68,10.87) | 2.51(2.45,2.57) |
| Croatia | 62.96(-8.81,155.63) | 2.56(-0.37,6.52) |  | 218.00(-28.62,557.49) | 3.08(-0.41,7.99) |  | 20.47(-3.31,48.50) | 1.28(1.14,1.41) |
| Cuba | 56.93(-7.72,148.28) | 1.03(-0.14,2.65) |  | 128.91(-18.40,330.94) | 1.29(-0.18,3.33) |  | 25.22(6.36,46.87) | 3.55(3.38,3.72) |
| Cyprus | 409.25(-51.69,1035.46) | 4.22(-0.54,10.61) |  | 928.68(-122.03,2365.79) | 4.40(-0.57,11.24) |  | 4.25(-7.24,18.20) | 2.60(2.54,2.66) |
| Czechia | 31.86(-4.27,84.35) | 5.46(-0.76,14.83) |  | 58.69(-8.25,149.12) | 3.26(-0.48,8.14) |  | -40.23(-54.62,-19.81) | 0.10(0.03,0.17) |
| C么te d'Ivoire | 170.64(-21.36,443.35) | 1.26(-0.16,3.29) |  | 403.74(-53.53,1049.18) | 1.71(-0.23,4.45) |  | 35.37(16.24,56.39) | 2.93(2.66,3.20) |
| Democratic People's Republic of Korea | 314.60(-43.01,826.80) | 2.62(-0.38,7.08) |  | 880.39(-121.76,2245.94) | 3.14(-0.45,8.21) |  | 19.87(-0.96,45.56) | 2.75(2.61,2.90) |
| Democratic Republic of the Congo | 243.97(-36.01,655.99) | 2.21(-0.32,5.98) |  | 750.15(-116.96,1984.56) | 2.98(-0.47,7.72) |  | 35.04(4.89,67.29) | 0.80(0.73,0.88) |
| Denmark | 144.86(-19.39,376.45) | 1.72(-0.23,4.50) |  | 288.67(-38.31,731.32) | 2.18(-0.29,5.51) |  | 26.82(12.65,42.61) | 1.60(1.20,2.00) |
| Djibouti | 2.68(-0.38,6.90) | 3.04(-0.44,7.89) |  | 10.95(-1.55,28.24) | 2.80(-0.41,6.99) |  | -8.02(-26.53,17.30) | 0.94(0.83,1.04) |
| Dominica | 2.07(-0.28,5.50) | 3.65(-0.49,9.65) |  | 2.61(-0.35,6.61) | 3.47(-0.47,8.83) |  | -4.91(-19.25,14.73) | 0.88(0.72,1.03) |
| Dominican Republic | 178.54(-23.33,452.94) | 6.09(-0.80,15.32) |  | 529.04(-75.29,1341.19) | 5.54(-0.79,14.02) |  | -9.03(-24.11,10.01) | 2.12(1.80,2.43) |
| Ecuador | 109.55(-13.85,285.45) | 2.43(-0.31,6.28) |  | 341.24(-43.95,894.11) | 2.26(-0.29,5.87) |  | -7.19(-19.03,6.64) | 1.96(1.80,2.11) |
| Egypt | 672.26(-93.87,1745.53) | 3.96(-0.57,10.02) |  | 1596.24(-201.67,3984.79) | 4.25(-0.56,10.44) |  | 7.27(-12.71,32.63) | 0.55(0.45,0.65) |
| El Salvador | 182.46(-24.78,460.91) | 6.50(-0.89,16.46) |  | 449.35(-59.73,1131.65) | 6.58(-0.87,16.56) |  | 1.12(-14.39,22.42) | 2.40(2.28,2.51) |
| Equatorial Guinea | 3.60(-0.59,9.36) | 2.43(-0.41,6.46) |  | 9.30(-1.46,24.37) | 2.68(-0.42,7.02) |  | 10.14(-25.16,59.06) | -0.99(-1.13,-0.84) |
| Eritrea | 21.65(-3.18,58.27) | 2.92(-0.44,7.81) |  | 48.27(-7.14,125.88) | 2.87(-0.42,7.73) |  | -1.64(-27.65,45.52) | 0.23(0.15,0.31) |
| Estonia | 16.09(-1.98,41.77) | 0.81(-0.10,2.12) |  | 39.55(-5.16,99.36) | 1.26(-0.16,3.16) |  | 55.32(35.75,78.75) | 3.50(3.41,3.59) |
| Eswatini | 6.13(-0.89,16.10) | 2.75(-0.41,7.35) |  | 11.02(-1.50,28.61) | 2.70(-0.37,7.12) |  | -1.86(-22.35,25.31) | 0.95(0.69,1.20) |
| Ethiopia | 489.64(-73.08,1272.66) | 3.50(-0.53,9.50) |  | 1152.49(-159.68,3072.09) | 3.46(-0.49,9.24) |  | -1.29(-20.60,21.28) | 0.41(0.29,0.54) |
| Fiji | 3.36(-0.42,8.59) | 1.37(-0.18,3.53) |  | 7.22(-0.93,18.42) | 1.35(-0.18,3.47) |  | -1.52(-19.20,21.29) | 1.99(1.91,2.08) |
| Finland | 90.25(-12.62,230.56) | 1.26(-0.18,3.22) |  | 147.96(-19.96,375.66) | 1.01(-0.13,2.58) |  | -20.10(-31.10,-7.18) | 1.04(0.79,1.28) |
| France | 2258.74(-298.40,6061.07) | 2.57(-0.34,6.83) |  | 3609.05(-498.43,9350.05) | 2.03(-0.28,5.32) |  | -20.87(-28.96,-12.30) | 1.02(0.93,1.10) |
| Gabon | 11.79(-1.75,31.44) | 2.45(-0.37,6.59) |  | 18.80(-2.62,48.47) | 2.54(-0.36,6.60) |  | 3.66(-18.42,29.32) | -0.56(-0.63,-0.50) |
| Gambia | 6.76(-0.96,17.60) | 2.74(-0.39,7.16) |  | 25.23(-3.58,62.78) | 3.55(-0.51,8.93) |  | 29.53(0.12,69.79) | 1.46(1.25,1.68) |
| Georgia | 88.42(-11.48,231.65) | 1.48(-0.19,3.87) |  | 194.61(-26.30,484.35) | 3.07(-0.41,7.64) |  | 106.58(71.96,163.04) | 4.15(3.86,4.44) |
| Germany | 1775.56(-228.17,4687.05) | 1.36(-0.17,3.58) |  | 4364.02(-577.37,11111.06) | 1.92(-0.25,4.94) |  | 40.87(22.21,61.34) | 3.17(2.81,3.53) |
| Ghana | 78.96(-11.00,199.64) | 1.85(-0.26,4.63) |  | 218.01(-27.85,575.32) | 1.96(-0.26,5.14) |  | 6.04(-14.70,33.81) | 0.36(0.14,0.58) |
| Greece | 352.59(-47.52,930.86) | 2.39(-0.32,6.26) |  | 775.06(-105.76,1984.40) | 2.57(-0.34,6.61) |  | 7.81(-3.72,21.05) | 2.61(2.48,2.74) |
| Greenland | 0.62(-0.08,1.63) | 2.51(-0.32,6.50) |  | 1.19(-0.15,3.06) | 2.16(-0.28,5.65) |  | -13.88(-27.35,2.34) | 2.33(2.23,2.43) |
| Grenada | 3.51(-0.49,9.29) | 4.35(-0.61,11.44) |  | 4.46(-0.57,11.64) | 4.80(-0.63,12.43) |  | 10.29(-6.96,30.37) | -0.01(-0.19,0.17) |
| Guam | 0.45(-0.06,1.17) | 0.96(-0.12,2.45) |  | 1.21(-0.15,3.17) | 0.55(-0.07,1.46) |  | -42.85(-52.08,-34.40) | 3.05(2.90,3.20) |
| Guatemala | 168.10(-21.88,431.12) | 7.01(-0.95,18.13) |  | 620.04(-83.66,1585.26) | 6.50(-0.89,16.46) |  | -7.28(-18.19,4.67) | 2.42(2.28,2.56) |
| Guinea | 73.65(-11.07,190.72) | 2.77(-0.43,7.29) |  | 159.03(-22.58,383.29) | 3.76(-0.54,9.16) |  | 35.63(10.54,76.20) | 0.03(-0.32,0.38) |
| Guinea-Bissau | 8.01(-1.25,20.91) | 2.94(-0.47,7.62) |  | 15.57(-2.14,40.67) | 3.69(-0.54,9.75) |  | 25.40(-0.39,62.24) | -0.19(-0.26,-0.11) |
| Guyana | 13.49(-1.73,33.97) | 4.24(-0.55,10.68) |  | 24.39(-3.27,63.61) | 4.77(-0.65,12.22) |  | 12.54(-2.20,33.48) | 2.32(2.19,2.45) |
| Haiti | 177.37(-28.52,458.75) | 7.45(-1.20,19.14) |  | 393.28(-54.63,995.16) | 7.79(-1.10,19.87) |  | 4.66(-15.59,28.35) | 0.36(0.30,0.43) |
| Honduras | 105.76(-14.59,262.06) | 6.31(-0.89,15.45) |  | 433.21(-60.98,1071.31) | 8.85(-1.27,21.78) |  | 40.30(8.58,79.04) | 2.19(2.02,2.36) |
| Hungary | 188.12(-24.38,498.75) | 1.33(-0.17,3.49) |  | 291.69(-39.45,736.12) | 1.38(-0.19,3.50) |  | 4.01(-10.12,22.41) | 1.81(1.66,1.96) |
| Iceland | 6.69(-0.85,17.84) | 2.21(-0.28,5.91) |  | 15.49(-2.09,39.10) | 2.31(-0.31,5.86) |  | 4.46(-8.95,19.69) | 1.79(1.64,1.94) |
| India | 10826.28(-1496.61,27839.58) | 3.09(-0.43,7.98) |  | 39509.42(-5576.83,100784.23) | 4.05(-0.58,10.33) |  | 31.10(11.14,53.28) | 2.75(2.66,2.83) |
| Indonesia | 2097.11(-276.79,5430.18) | 2.66(-0.37,6.94) |  | 5641.64(-789.00,14156.96) | 3.36(-0.49,8.31) |  | 26.55(9.74,52.23) | 1.73(1.56,1.91) |
| Iran (Islamic Republic of) | 674.05(-90.59,1695.47) | 3.84(-0.54,9.79) |  | 2604.42(-350.40,6539.97) | 4.04(-0.55,10.12) |  | 5.23(-11.50,26.23) | 3.38(3.24,3.52) |
| Iraq | 178.87(-23.86,463.24) | 2.46(-0.33,6.34) |  | 502.37(-65.54,1260.06) | 3.03(-0.41,7.55) |  | 23.10(-3.69,69.87) | 0.65(0.48,0.82) |
| Ireland | 101.64(-13.83,262.23) | 2.54(-0.34,6.55) |  | 175.89(-23.95,456.63) | 2.07(-0.28,5.36) |  | -18.44(-28.54,-7.45) | 0.52(0.33,0.70) |
| Israel | 91.41(-11.45,241.09) | 1.97(-0.25,5.18) |  | 275.70(-38.35,723.69) | 2.05(-0.28,5.37) |  | 4.03(-10.85,21.59) | 1.62(1.41,1.83) |
| Italy | 2063.27(-278.31,5284.76) | 2.34(-0.31,5.99) |  | 4331.87(-578.60,11319.08) | 2.33(-0.30,6.18) |  | -0.37(-10.79,11.94) | 2.21(2.10,2.32) |
| Jamaica | 76.42(-10.26,195.12) | 4.15(-0.56,10.61) |  | 142.25(-18.66,359.98) | 4.20(-0.54,10.62) |  | 1.16(-13.43,21.43) | 1.45(1.31,1.58) |
| Japan | 1676.76(-213.18,4317.74) | 1.03(-0.13,2.65) |  | 3451.69(-445.55,8806.75) | 0.73(-0.09,1.85) |  | -29.65(-34.24,-25.24) | 2.05(1.86,2.24) |
| Jordan | 19.60(-2.68,51.35) | 2.02(-0.28,5.33) |  | 96.65(-12.79,252.33) | 1.93(-0.27,4.91) |  | -4.75(-23.06,14.32) | 1.11(0.93,1.30) |
| Kazakhstan | 97.36(-12.22,261.14) | 0.86(-0.11,2.29) |  | 150.19(-18.93,400.18) | 1.00(-0.13,2.62) |  | 16.50(-5.42,43.85) | 0.64(0.44,0.84) |
| Kenya | 148.34(-20.02,395.55) | 2.32(-0.32,6.11) |  | 345.81(-46.81,889.70) | 2.20(-0.31,5.78) |  | -5.13(-20.75,14.78) | 0.24(0.20,0.28) |
| Kiribati | 0.30(-0.04,0.78) | 1.16(-0.15,2.98) |  | 0.60(-0.08,1.58) | 1.23(-0.16,3.32) |  | 6.47(-9.62,26.28) | 0.52(0.48,0.56) |
| Kuwait | 6.91(-0.88,17.90) | 1.56(-0.20,4.00) |  | 34.35(-4.55,86.93) | 1.62(-0.22,4.18) |  | 3.64(-9.44,19.03) | 1.60(1.11,2.10) |
| Kyrgyzstan | 29.21(-3.66,78.76) | 1.09(-0.14,2.93) |  | 55.81(-7.16,146.85) | 1.42(-0.19,3.72) |  | 30.35(12.95,48.97) | 0.64(0.56,0.71) |
| Lao People's Democratic Republic | 47.90(-6.67,123.70) | 3.09(-0.44,8.04) |  | 132.51(-17.16,344.55) | 3.93(-0.52,10.23) |  | 27.16(4.84,56.01) | 1.44(1.41,1.47) |
| Latvia | 28.37(-3.67,73.62) | 0.81(-0.10,2.09) |  | 53.05(-7.23,135.97) | 1.19(-0.16,3.00) |  | 47.41(25.70,74.53) | 3.58(3.46,3.70) |
| Lebanon | 35.26(-5.40,94.60) | 2.04(-0.32,5.54) |  | 106.40(-13.52,276.75) | 1.59(-0.20,4.16) |  | -22.03(-47.06,20.19) | 1.55(1.38,1.73) |
| Lesotho | 16.05(-2.20,41.59) | 2.21(-0.31,5.73) |  | 25.27(-3.36,66.06) | 3.02(-0.41,7.79) |  | 36.71(4.54,67.55) | 1.00(0.72,1.28) |
| Liberia | 22.31(-3.11,56.35) | 2.57(-0.36,6.68) |  | 47.04(-6.66,121.52) | 3.34(-0.48,8.53) |  | 29.78(5.79,64.17) | -1.11(-1.36,-0.86) |
| Libya | 35.35(-4.77,89.59) | 2.16(-0.30,5.43) |  | 89.64(-12.71,229.08) | 2.24(-0.32,5.69) |  | 3.48(-20.01,38.43) | 1.50(1.45,1.55) |
| Lithuania | 38.42(-5.07,101.38) | 0.86(-0.11,2.28) |  | 77.53(-10.48,199.87) | 1.19(-0.16,3.11) |  | 38.18(21.74,58.08) | 3.49(3.35,3.64) |
| Luxembourg | 11.60(-1.61,29.08) | 2.14(-0.30,5.33) |  | 23.55(-3.23,58.22) | 1.97(-0.27,4.88) |  | -8.26(-20.13,4.80) | 0.83(0.68,0.98) |
| Madagascar | 134.54(-19.29,346.87) | 3.52(-0.51,9.07) |  | 232.09(-31.49,586.84) | 3.36(-0.46,8.50) |  | -4.57(-23.99,20.17) | -1.09(-1.28,-0.89) |
| Malawi | 68.37(-10.17,180.44) | 2.47(-0.37,6.52) |  | 145.72(-20.60,365.19) | 2.72(-0.40,6.78) |  | 9.99(-8.54,36.94) | -0.01(-0.22,0.19) |
| Malaysia | 145.10(-19.00,378.80) | 1.73(-0.23,4.50) |  | 541.64(-69.45,1394.17) | 2.32(-0.30,5.94) |  | 33.81(15.22,62.86) | 2.33(2.26,2.39) |
| Maldives | 1.47(-0.19,3.71) | 2.48(-0.34,6.30) |  | 6.59(-0.82,17.57) | 2.43(-0.30,6.19) |  | -2.29(-23.08,26.20) | 2.10(1.90,2.31) |
| Mali | 75.01(-11.30,198.48) | 2.64(-0.42,6.78) |  | 205.37(-29.11,523.85) | 3.26(-0.47,8.09) |  | 23.84(0.24,51.04) | 0.15(0.08,0.23) |
| Malta | 19.07(-2.49,47.61) | 4.70(-0.62,11.64) |  | 45.94(-6.61,117.66) | 4.13(-0.59,10.54) |  | -12.16(-25.17,0.35) | 2.60(2.46,2.74) |
| Marshall Islands | 0.22(-0.03,0.59) | 1.89(-0.25,5.12) |  | 0.41(-0.06,1.07) | 1.93(-0.27,5.05) |  | 2.40(-15.16,23.22) | 1.50(1.33,1.66) |
| Mauritania | 15.42(-2.10,41.29) | 2.01(-0.28,5.43) |  | 39.96(-5.36,101.57) | 2.46(-0.34,6.22) |  | 22.28(-4.18,57.19) | 0.60(0.58,0.63) |
| Mauritius | 9.35(-1.23,24.45) | 1.62(-0.22,4.23) |  | 28.59(-3.72,74.93) | 1.68(-0.22,4.40) |  | 3.73(-6.62,13.25) | 3.06(2.95,3.18) |
| Mexico | 1693.40(-219.32,4347.11) | 4.92(-0.64,12.49) |  | 5167.03(-722.07,12837.49) | 4.52(-0.64,11.21) |  | -8.13(-14.82,-0.47) | 2.39(2.27,2.52) |
| Micronesia (Federated States of) | 0.66(-0.09,1.75) | 1.69(-0.23,4.48) |  | 0.94(-0.13,2.48) | 1.81(-0.25,4.73) |  | 7.07(-11.82,29.36) | 1.26(1.17,1.34) |
| Monaco | 1.16(-0.15,3.08) | 1.47(-0.19,3.90) |  | 1.74(-0.22,4.51) | 1.48(-0.19,3.87) |  | 0.52(-17.91,22.84) | 0.45(0.16,0.74) |
| Mongolia | 16.23(-2.00,42.26) | 1.77(-0.22,4.64) |  | 28.90(-3.59,76.97) | 1.67(-0.21,4.47) |  | -5.75(-21.39,10.17) | 0.34(0.24,0.44) |
| Montenegro | 13.85(-1.94,34.94) | 2.38(-0.33,5.97) |  | 31.33(-4.47,80.23) | 3.61(-0.51,9.29) |  | 51.91(18.40,98.63) | 3.01(2.88,3.14) |
| Morocco | 231.70(-32.67,610.49) | 1.97(-0.28,5.20) |  | 649.09(-92.09,1617.23) | 2.41(-0.35,6.03) |  | 22.11(-3.24,58.17) | 2.22(2.13,2.31) |
| Mozambique | 153.78(-23.46,410.78) | 3.59(-0.56,9.80) |  | 313.48(-51.55,793.90) | 4.10(-0.70,10.31) |  | 14.40(-10.06,44.20) | -0.33(-0.44,-0.21) |
| Myanmar | 505.09(-68.78,1325.62) | 2.83(-0.40,7.39) |  | 1357.43(-171.96,3441.57) | 3.48(-0.45,8.72) |  | 23.16(-1.07,55.40) | 2.07(1.98,2.16) |
| Namibia | 9.59(-1.26,24.95) | 1.93(-0.26,5.00) |  | 23.22(-3.17,58.64) | 2.24(-0.31,5.71) |  | 15.98(-3.65,42.19) | 1.08(0.98,1.17) |
| Nauru | 0.04(-0.01,0.11) | 1.42(-0.19,3.61) |  | 0.06(-0.01,0.16) | 1.65(-0.26,4.54) |  | 15.98(-13.26,78.79) | 0.92(0.78,1.06) |
| Nepal | 272.51(-39.64,703.13) | 3.98(-0.60,10.46) |  | 1000.14(-143.64,2525.84) | 5.59(-0.82,14.23) |  | 40.35(16.15,74.72) | 3.01(2.86,3.16) |
| Netherlands | 488.54(-67.53,1274.53) | 2.39(-0.33,6.23) |  | 793.19(-112.46,1986.72) | 2.00(-0.28,5.02) |  | -16.18(-24.80,-7.29) | 0.93(0.85,1.01) |
| New Zealand | 128.73(-17.60,334.28) | 3.39(-0.47,8.73) |  | 331.79(-45.60,820.43) | 3.62(-0.50,8.96) |  | 6.90(-3.60,16.48) | 1.87(1.79,1.95) |
| Nicaragua | 68.52(-8.90,173.31) | 5.32(-0.70,13.46) |  | 182.99(-24.66,472.10) | 4.42(-0.60,11.16) |  | -16.98(-28.26,-3.76) | 1.57(1.32,1.82) |
| Niger | 56.93(-8.16,149.49) | 3.03(-0.46,8.16) |  | 211.61(-30.88,534.02) | 3.88(-0.58,9.90) |  | 27.89(5.58,55.75) | 0.57(0.52,0.63) |
| Nigeria | 659.11(-92.96,1746.27) | 2.01(-0.29,5.31) |  | 1281.00(-183.79,3323.12) | 2.03(-0.30,5.19) |  | 1.10(-18.50,30.26) | -1.21(-1.34,-1.07) |
| Niue | 0.03(-0.00,0.08) | 1.21(-0.16,3.13) |  | 0.02(-0.00,0.06) | 1.09(-0.14,2.80) |  | -9.86(-25.54,6.17) | 0.26(0.07,0.46) |
| North Macedonia | 27.04(-3.52,73.99) | 1.63(-0.21,4.51) |  | 63.58(-8.54,172.67) | 2.38(-0.33,6.33) |  | 45.78(16.52,81.77) | 2.53(2.35,2.71) |
| Northern Mariana Islands | 0.08(-0.01,0.22) | 0.84(-0.11,2.16) |  | 0.34(-0.04,0.88) | 0.91(-0.12,2.36) |  | 7.93(-10.87,26.66) | 4.85(4.35,5.35) |
| Norway | 146.49(-19.54,369.31) | 1.99(-0.26,5.02) |  | 218.44(-29.31,563.99) | 1.88(-0.25,4.91) |  | -5.33(-15.05,3.98) | 0.35(0.07,0.62) |
| Oman | 13.34(-1.87,34.65) | 2.58(-0.36,6.64) |  | 31.62(-4.59,80.27) | 2.60(-0.37,6.56) |  | 0.55(-27.89,38.88) | 0.16(-0.07,0.39) |
| Pakistan | 1664.08(-239.46,4311.11) | 3.59(-0.53,9.23) |  | 4153.94(-568.96,10201.69) | 4.88(-0.68,12.00) |  | 35.96(15.87,67.00) | 0.33(0.14,0.51) |
| Palau | 0.05(-0.01,0.14) | 0.72(-0.10,1.87) |  | 0.11(-0.01,0.29) | 0.70(-0.09,1.78) |  | -3.26(-18.13,11.75) | 1.90(1.73,2.07) |
| Palestine | 26.88(-3.79,69.31) | 4.05(-0.59,10.62) |  | 61.17(-8.58,158.43) | 3.93(-0.57,10.38) |  | -2.93(-22.36,23.98) | -0.34(-0.44,-0.24) |
| Panama | 48.35(-6.12,123.62) | 3.57(-0.45,9.05) |  | 165.44(-22.19,410.29) | 3.61(-0.49,8.98) |  | 1.24(-12.95,18.09) | 2.16(2.02,2.29) |
| Papua New Guinea | 16.46(-2.26,42.80) | 1.32(-0.19,3.46) |  | 51.72(-7.03,136.82) | 1.54(-0.22,4.12) |  | 16.59(-6.41,41.84) | 0.58(0.51,0.66) |
| Paraguay | 61.55(-8.07,162.28) | 3.05(-0.40,8.03) |  | 188.64(-23.76,485.32) | 3.55(-0.45,9.19) |  | 16.50(-3.98,45.32) | 1.96(1.86,2.06) |
| Peru | 273.39(-35.91,728.54) | 2.52(-0.33,6.70) |  | 924.29(-118.69,2412.62) | 2.80(-0.36,7.31) |  | 10.83(-5.41,35.62) | 2.55(2.44,2.66) |
| Philippines | 341.08(-42.91,884.83) | 1.60(-0.20,4.09) |  | 1273.54(-162.49,3261.11) | 1.96(-0.25,5.02) |  | 22.81(8.90,37.73) | 2.33(2.25,2.42) |
| Poland | 984.61(-123.57,2457.89) | 2.38(-0.30,5.97) |  | 1881.31(-242.42,4843.12) | 2.43(-0.31,6.28) |  | 2.12(-10.56,14.45) | 2.13(1.71,2.54) |
| Portugal | 490.94(-65.80,1268.08) | 3.74(-0.51,9.59) |  | 929.09(-133.12,2349.72) | 3.09(-0.44,7.83) |  | -17.32(-26.42,-5.94) | 1.94(1.84,2.05) |
| Puerto Rico | 72.09(-9.09,186.97) | 2.16(-0.27,5.61) |  | 149.34(-19.22,381.61) | 1.71(-0.22,4.37) |  | -21.16(-30.87,-10.98) | 2.42(2.28,2.55) |
| Qatar | 1.13(-0.15,2.97) | 2.07(-0.29,5.62) |  | 6.39(-0.74,17.33) | 1.43(-0.19,3.92) |  | -30.90(-46.58,-11.32) | -1.30(-1.86,-0.73) |
| Republic of Korea | 574.33(-69.96,1561.45) | 2.33(-0.29,6.23) |  | 1933.57(-267.98,5032.39) | 2.06(-0.29,5.38) |  | -11.49(-39.99,10.95) | 3.88(3.68,4.09) |
| Republic of Moldova | 42.95(-5.41,113.28) | 1.14(-0.14,2.99) |  | 90.42(-12.85,234.91) | 1.49(-0.21,3.87) |  | 31.16(16.54,49.94) | 3.16(3.01,3.32) |
| Romania | 351.93(-44.44,917.19) | 1.43(-0.18,3.72) |  | 523.55(-74.77,1317.97) | 1.28(-0.18,3.21) |  | -10.45(-25.45,9.25) | 1.94(1.78,2.11) |
| Russian Federation | 1519.27(-194.45,3955.10) | 0.93(-0.12,2.41) |  | 3234.97(-408.01,8329.98) | 1.32(-0.17,3.40) |  | 42.29(26.14,58.53) | 2.61(2.43,2.79) |
| Rwanda | 68.48(-9.76,176.67) | 3.36(-0.49,8.61) |  | 113.22(-15.72,293.77) | 2.59(-0.37,6.79) |  | -22.85(-42.56,-1.34) | -0.82(-1.26,-0.37) |
| Saint Kitts and Nevis | 1.39(-0.19,3.60) | 3.94(-0.54,10.15) |  | 1.75(-0.23,4.52) | 3.52(-0.48,9.09) |  | -10.65(-22.66,1.45) | -0.45(-0.59,-0.32) |
| Saint Lucia | 5.07(-0.69,12.74) | 7.07(-0.97,17.59) |  | 11.37(-1.59,28.11) | 4.98(-0.70,12.29) |  | -29.49(-39.28,-18.44) | 1.66(1.47,1.86) |
| Saint Vincent and the Grenadines | 4.48(-0.62,11.15) | 6.88(-0.95,17.00) |  | 7.65(-1.01,19.50) | 6.10(-0.82,15.43) |  | -11.37(-20.60,-0.36) | 1.73(1.58,1.88) |
| Samoa | 0.99(-0.13,2.63) | 1.52(-0.19,3.96) |  | 1.67(-0.21,4.33) | 1.44(-0.18,3.72) |  | -5.34(-19.22,11.94) | 0.97(0.92,1.03) |
| San Marino | 0.64(-0.09,1.71) | 1.73(-0.23,4.58) |  | 1.09(-0.15,2.91) | 1.15(-0.15,3.20) |  | -33.51(-45.19,-17.53) | 1.01(0.70,1.32) |
| Sao Tome and Principe | 1.04(-0.15,2.71) | 1.91(-0.28,4.94) |  | 2.16(-0.31,5.52) | 2.76(-0.42,6.99) |  | 44.86(11.72,84.70) | 0.52(0.45,0.59) |
| Saudi Arabia | 103.12(-14.02,270.87) | 2.30(-0.31,5.96) |  | 262.74(-35.65,681.32) | 2.42(-0.34,6.36) |  | 5.29(-16.88,33.61) | 0.02(-0.13,0.18) |
| Senegal | 58.71(-8.68,152.01) | 2.44(-0.37,6.30) |  | 154.22(-21.74,396.91) | 2.77(-0.40,7.25) |  | 13.33(-12.14,49.54) | 0.84(0.79,0.89) |
| Serbia | 140.61(-18.72,368.81) | 1.58(-0.22,4.12) |  | 261.35(-37.67,685.31) | 1.50(-0.21,3.92) |  | -5.16(-20.46,18.92) | 2.58(2.45,2.72) |
| Seychelles | 0.74(-0.09,1.98) | 1.33(-0.17,3.59) |  | 1.41(-0.18,3.84) | 1.45(-0.19,3.80) |  | 8.80(-5.49,28.26) | 0.95(0.84,1.06) |
| Sierra Leone | 35.87(-5.18,90.82) | 2.12(-0.31,5.30) |  | 77.96(-11.53,200.82) | 2.79(-0.42,7.30) |  | 31.81(3.87,65.33) | -0.35(-0.58,-0.12) |
| Singapore | 40.75(-5.07,106.17) | 2.03(-0.25,5.33) |  | 112.72(-13.79,305.38) | 1.34(-0.16,3.65) |  | -33.99(-41.22,-28.06) | 0.87(0.65,1.10) |
| Slovakia | 101.45(-13.81,265.40) | 1.74(-0.24,4.58) |  | 196.30(-25.92,486.51) | 2.02(-0.27,5.02) |  | 15.67(-6.64,40.66) | 2.12(1.97,2.26) |
| Slovenia | 25.50(-3.12,67.61) | 1.05(-0.13,2.76) |  | 58.11(-7.80,146.16) | 1.14(-0.15,2.92) |  | 9.25(-8.00,29.06) | 2.73(2.44,3.01) |
| Solomon Islands | 2.36(-0.33,6.34) | 2.76(-0.40,7.38) |  | 6.45(-0.93,16.18) | 2.82(-0.39,7.39) |  | 2.21(-15.61,24.88) | 0.97(0.92,1.02) |
| Somalia | 62.18(-10.09,175.32) | 3.91(-0.66,10.90) |  | 152.12(-24.09,397.04) | 3.75(-0.59,9.87) |  | -4.22(-20.71,17.79) | -0.48(-0.58,-0.38) |
| South Africa | 251.76(-31.45,672.34) | 1.40(-0.18,3.73) |  | 695.30(-88.50,1836.37) | 1.86(-0.24,4.87) |  | 32.36(10.55,57.84) | 1.99(1.78,2.21) |
| South Sudan | 63.25(-9.48,169.91) | 3.07(-0.47,8.23) |  | 75.41(-10.95,193.21) | 2.85(-0.41,7.16) |  | -7.25(-25.58,14.55) | -1.40(-1.69,-1.11) |
| Spain | 1740.83(-232.88,4468.35) | 3.21(-0.43,8.23) |  | 3772.20(-509.29,9489.59) | 3.09(-0.41,7.80) |  | -3.67(-17.09,8.92) | 1.91(1.67,2.15) |
| Sri Lanka | 90.18(-11.47,240.92) | 1.08(-0.14,2.87) |  | 312.75(-37.81,821.91) | 1.33(-0.16,3.40) |  | 22.85(0.65,50.81) | 3.27(3.10,3.45) |
| Sudan | 228.32(-35.60,596.22) | 3.15(-0.50,8.34) |  | 520.14(-74.59,1305.58) | 3.55(-0.53,8.78) |  | 12.52(-11.84,49.40) | 0.07(-0.02,0.15) |
| Suriname | 9.91(-1.27,24.65) | 4.34(-0.56,10.81) |  | 23.52(-3.42,62.34) | 4.07(-0.60,10.68) |  | -6.19(-22.26,13.18) | 1.57(1.46,1.68) |
| Sweden | 201.85(-26.84,529.79) | 1.24(-0.16,3.24) |  | 592.46(-77.55,1556.26) | 2.27(-0.29,6.02) |  | 83.52(64.53,105.21) | 2.99(2.73,3.24) |
| Switzerland | 139.54(-18.76,360.60) | 1.25(-0.17,3.23) |  | 286.62(-39.17,726.84) | 1.30(-0.18,3.34) |  | 3.90(-10.44,17.95) | 1.81(1.69,1.92) |
| Syrian Arab Republic | 137.91(-17.72,358.45) | 3.37(-0.44,8.70) |  | 323.81(-42.64,823.00) | 3.64(-0.51,9.17) |  | 7.96(-17.86,41.42) | 2.34(1.64,3.04) |
| Taiwan (Province of China) | 355.30(-45.60,919.54) | 2.92(-0.38,7.42) |  | 1107.21(-143.81,2890.96) | 2.46(-0.32,6.44) |  | -15.73(-22.88,-7.89) | 3.10(2.79,3.40) |
| Tajikistan | 31.12(-3.98,82.53) | 1.27(-0.16,3.34) |  | 65.87(-8.08,178.17) | 1.48(-0.19,3.97) |  | 16.94(0.60,35.49) | 0.31(0.20,0.42) |
| Thailand | 401.41(-54.36,1077.90) | 1.46(-0.20,3.94) |  | 1696.62(-226.12,4440.50) | 1.55(-0.21,4.05) |  | 6.11(-15.81,31.04) | 3.94(3.85,4.04) |
| Timor-Leste | 6.26(-0.80,16.10) | 3.11(-0.41,8.05) |  | 25.57(-3.36,66.51) | 3.88(-0.52,9.88) |  | 24.89(1.15,58.13) | 3.08(2.88,3.27) |
| Togo | 19.32(-2.75,49.52) | 2.21(-0.33,5.67) |  | 66.48(-8.92,167.41) | 2.77(-0.39,6.95) |  | 25.38(-0.86,60.98) | 1.24(1.21,1.27) |
| Tokelau | 0.02(-0.00,0.05) | 1.50(-0.20,3.91) |  | 0.02(-0.00,0.05) | 1.31(-0.17,3.29) |  | -12.94(-32.44,10.72) | 1.06(0.70,1.43) |
| Tonga | 0.63(-0.08,1.69) | 1.43(-0.19,3.83) |  | 0.96(-0.12,2.54) | 1.28(-0.17,3.38) |  | -10.46(-26.52,8.82) | 1.02(0.84,1.20) |
| Trinidad and Tobago | 15.21(-2.00,39.06) | 2.28(-0.30,5.92) |  | 34.43(-4.66,90.52) | 1.87(-0.26,4.87) |  | -17.98(-30.80,-3.75) | 2.05(1.95,2.15) |
| Tunisia | 92.99(-11.85,233.68) | 2.54(-0.34,6.58) |  | 323.43(-43.22,806.75) | 2.91(-0.39,7.20) |  | 14.61(-12.68,47.86) | 2.90(2.74,3.06) |
| Turkey | 496.63(-68.01,1271.69) | 1.79(-0.25,4.58) |  | 1312.18(-179.59,3336.44) | 1.63(-0.22,4.16) |  | -8.92(-29.35,15.25) | 2.30(1.97,2.63) |
| Turkmenistan | 16.42(-2.03,43.04) | 1.02(-0.13,2.69) |  | 44.59(-6.19,116.76) | 1.34(-0.19,3.49) |  | 31.34(11.41,55.85) | 2.23(2.15,2.31) |
| Tuvalu | 0.10(-0.01,0.27) | 2.16(-0.30,5.75) |  | 0.16(-0.02,0.40) | 1.97(-0.26,4.95) |  | -8.88(-24.87,7.67) | 0.44(0.22,0.66) |
| Uganda | 132.28(-21.02,339.46) | 2.65(-0.42,6.90) |  | 289.08(-43.85,733.97) | 2.64(-0.41,6.81) |  | -0.47(-19.96,27.76) | -0.53(-0.73,-0.34) |
| Ukraine | 553.18(-68.18,1452.44) | 0.83(-0.10,2.19) |  | 804.83(-103.60,2168.43) | 1.00(-0.13,2.68) |  | 20.26(2.04,43.59) | 1.80(1.67,1.93) |
| United Arab Emirates | 5.24(-0.73,13.59) | 1.77(-0.25,4.70) |  | 26.80(-3.33,70.36) | 1.74(-0.24,4.44) |  | -1.84(-26.96,22.57) | -0.71(-1.28,-0.14) |
| United Kingdom | 1444.62(-194.56,3687.00) | 1.53(-0.21,3.91) |  | 2481.51(-336.03,6290.19) | 1.64(-0.22,4.17) |  | 6.79(-0.57,11.76) | 1.36(1.21,1.51) |
| United Republic of Tanzania | 178.75(-25.87,463.46) | 2.27(-0.31,5.90) |  | 372.37(-54.70,986.51) | 1.92(-0.29,5.01) |  | -15.40(-32.23,11.02) | -0.56(-0.68,-0.45) |
| United States of America | 6586.57(-833.03,16856.63) | 1.98(-0.25,5.04) |  | 13403.82(-1800.56,34335.95) | 2.11(-0.28,5.41) |  | 6.39(-10.24,23.77) | 1.49(1.45,1.54) |
| United States Virgin Islands | 1.57(-0.20,4.15) | 2.43(-0.32,6.36) |  | 3.10(-0.40,8.03) | 1.75(-0.23,4.44) |  | -27.86(-40.06,-13.00) | 2.80(2.61,2.99) |
| Uruguay | 53.31(-6.48,136.96) | 1.37(-0.17,3.51) |  | 125.85(-16.42,323.14) | 1.97(-0.25,5.09) |  | 43.79(28.94,62.15) | 2.56(2.42,2.70) |
| Uzbekistan | 80.82(-10.31,218.43) | 0.76(-0.10,2.06) |  | 210.33(-25.37,557.09) | 1.00(-0.13,2.66) |  | 31.34(7.24,60.94) | 1.59(1.51,1.67) |
| Vanuatu | 1.03(-0.14,2.66) | 2.42(-0.35,6.25) |  | 2.88(-0.39,7.69) | 2.34(-0.33,6.23) |  | -3.46(-18.18,15.28) | 0.86(0.81,0.92) |
| Venezuela (Bolivarian Republic of) | 316.05(-40.35,814.38) | 3.79(-0.49,9.65) |  | 1161.02(-152.18,2852.11) | 4.17(-0.55,10.26) |  | 10.03(-8.94,28.71) | 2.73(2.57,2.90) |
| Viet Nam | 688.20(-91.24,1774.10) | 1.97(-0.26,5.11) |  | 2159.61(-277.61,5461.53) | 2.65(-0.35,6.74) |  | 34.86(11.28,65.41) | 2.52(2.45,2.60) |
| Yemen | 133.95(-20.85,348.58) | 3.87(-0.61,9.85) |  | 466.66(-71.31,1180.45) | 4.76(-0.75,12.13) |  | 22.94(-7.65,63.40) | 1.12(1.08,1.16) |
| Zambia | 63.56(-8.70,164.28) | 3.18(-0.44,8.26) |  | 160.21(-23.76,438.25) | 3.50(-0.51,9.20) |  | 10.09(-21.63,56.89) | 0.13(-0.04,0.31) |
| Zimbabwe | 72.41(-9.26,182.03) | 2.32(-0.30,5.80) |  | 130.53(-17.29,335.69) | 2.65(-0.37,6.82) |  | 14.31(-11.40,37.14) | 0.43(0.14,0.72) |
